# Supplementary material for: Circulating MicroRNAs and Extracellular Vesicle-Derived MicroRNAs as Predictors of Functional Recovery in Ischemic Stroke Patients: A Systematic Review and Meta-Analysis
Source: Int J Mol Sci. 2022 Dec 23;24(1):251. doi: 10.3390/ijms24010251 (PMC9820088; doi:10.3390/ijms24010251)
Supplement: Supplementary file 1 [file ijms-24-00251-s001.zip › Supplimental S1.pdf]

Table S1. Demographic data of included studies

Study +ref	No patients recruited (stroke + controls)	Inclusion criteria	Exclusion criteria	Demographic data (age/ sex) (stroke patients vs controls)	Differing comorbidities and lab parameters (p<0.05)	Disability scale used	Score mean at admission
Ma et al, 2022 [1]	188 (72 AIS, 60 patients with high risk of stroke and 56 healthy controls)	-AIS diagnosis (clinical + CT/MRI)	- infectious disease;<br>-history of autoimmune disease or cancer;<br>-peripheral vascular disease, cerebral hemorrhage, transient ischemic attack, cardiac dysfunction.	AIS: 69.54±13.69; At risk subjects: 68.64±10.14; Controls: 66.36±11.19<br>M:F= 43/29	Hypertension, homocysteinemia, intima – media thickness, carotid atherosclerosis,	NIHSS	NIHSS <7, 40 (55.56%). NIHSS ≥ 7 32 (44.44%).
Peng et al., 2015 [2]	124 (72 AIS + 52 controls)	- first stroke Diagnosis (clinical + CT/MRI)<br>-ages 55 to 75<br>-patient arrival at the hospital after 4.5 h but within 24 h<br>- NIHSS score 4 to 15,<br>- without hemorrhagic transformation	-recurrent stroke, tumors, abnormal renal or liver function, infectious diseases, immune diseases, blood disorders, psychiatric illness	AIS: 72.4 (9.2) vs controls: 70.7 (7.5)<br>M:F=41/31	No significant differences	NIHSS	8.4±2.2
Wu et al., 2017 [3]	181 (50 AIS, 81 TIA patients and 50 healthy controls)	- focal neurological deficit lasting for more than 24 h<br>-AIS diagnosis (clinical + CT/MRI)	-history of hemorrhagic infarction, peripheral arterial occlusive diseases, chronic liver/kidney disease, cancer	AIS: 60.1 (11.3) vs TIA 60.8 (11.3) vs Controls 58.3 (9.5)<br>M:F=160:67	Hypertension Glucose, TG, HDL-C, LDL-C Antihypertensive drug use, Previous stroke	NIHSS on admission<br><br>BI at discharge<br><br>mRS at discharge	- 14.0 (10.0-17.0)<br><br>- 40.0 (25.0-85.0)<br><br>- 4.0 (3.0-4.0)
Kijpaisalratana et al. 2020 [4]	23 AIS + central vertigo + 35 peripheral	-age ≥45<br>- acute vertigo	-Altered state of consciousness,<br>-BT > 100.4 °F,	AIS with central vertigo: 64.52 (11.80) vs Peripheral	Diabetes mellitus, History of myocardial infarction,	NIHSS	3 (2-6)

	vertigo without AIS	-diagnosis of central vertigo caused by ischemic stroke<br>-hospital admission within 72 h of onset	-WBC > 15,000 cell/µL,<br>-Serum creatinine > 2 mg/dl,<br>-Liver enzymes > 3 fold of upper normal limit, cirrhosis, active malignancy or autoimmune disease,<br>-concurrent intracranial pathology.	Vertigo: 63.69 (9.42)<br>AIS, M:F=18:5	Smoking, Hemoglobin, WBC count, Creatinine, Fasting blood sugar, HbA1C.		
Kautzky et al., 2022 [5]	80 (40 AIS+40 Controls)	AIS diagnosis (clinical and imaging) within 24 h of onset	Infection, inflammatory disease, active malignancy, major surgery in the last 3 months, heparin treatment in the last 4 weeks, head trauma I the last 2 weeks	Stroke:74.7±13.8<br>Controls: 70.2±8.7;<br>Stroke: 55%<br>Controls: 40%	Atrial fibrillation, peripheral artery disease, previous stroke<br>CRP, AST, glucose, monocytes<br>Albumin, total cholesterol, LDL, HDL, TG, hematocrit	NIHSS	6.7±5.7
Zhu et al., 2019 [6]	340 (170 AIS + 170 controls)	- first AIS diagnosis (clinical +C T/MRI)<br>- age >18 years old;<br>- admission within 24 hours of onset	- intracranial hemorrhage, severe infection, inflammatory disease, malignancy, immunosuppressive treatment, pregnancy, lactation.	-AIS: 64.9 ± 12.5 vs Controls: 63.9 ± 10.5<br>M:F= 124:46	No significant differences	NIHSS	7.9 ± 3.4
Niu et al., 2021 [7]	596 (453 AIS + 143 controls)	- admission within 72 h of onset<br>- AIS diagnosis (clinical +C T/CTA/MRI/MRA)	NR	LAA (193): 62.9 (10.5), SAO (155) 62.9 (11.8) As (105): 63.4 (9.6)<br>vs Controls (143) 64.7 (8.9)<br>AIS, M:F=241:212	No significant differences	NIHSS	-

Li et al., 2022 [8]	92 (50 AIS, 42 controls)	-AIS diagnosis (clinical + DWI-MRI)<br>-first ischemic stroke<br>-age 18 – 80<br>-NIHSS<25 points<br>- admission within 6 h	NR	AIS patients: 18– 80 years	No significant differences	NIHSS	
Kotb 2019 et al., [9]	44 stroke patients + 22 age and sex matched controls	- age ≥ 18<br>- admission within 24 h of onset<br>- thrombotic stroke, anterior circulation	- Intracerebral hemorrhage, hemorrhagic infarction,<br>- Extensive infarction, anticoagulant or thrombolytic treatment<br>-Other neurological disorders	63.69, 6.42 (62.50, 54.00) vs 81.00 +- 64.38 -6.23 (65.00,56)	Cholesterol, Hemoglobin, Platelets, Urea, Serum creatinine	GCS- short-term prognosis of ACI	**NA**
Wu et al., 2020 [10]	112 ACI + 112 controls	- first ischemic stroke within 6 hours after the symptom onset<br>- CT/MRI showing focal neurological deficits	- cancer, renal, cardiovascular disease, autoimmune disease	64.56 ± 6.03 vs 63.42 ± 5.71<br>M:F=68/44 vs 70/42	BMI, Hypertension, Hyperlipidemia, Diabetes	GOS- short-term prognosis of ACI	**NA**
Huang et al., 2016 [11]	76 IS patients (39 PSCI + 37 PSCN) vs 38 age-matched controls	- history of stroke and/or CT/MRI consistent with cerebrovascular disease<br>- cognitive impairment judged due to a vascular cause<br>- MoCA score <26<br>- first stroke event, and within 1 year<br>- conscious with stable vital signs	- other causes of cognitive impairment, long duration of cognitive impairment, treatment for cognitive impairment<br>- serious speech, vision, hearing impairment or psychiatric disorders, alcohol or drug abuse<br>- BDI Score > 13<br>- heart failure, respiratory failure, or other organ failure;	66.2±88 vs 65.6±7.4<br>M:F=20/19	Diabetes, Hypertension	MoCA score	7.6±5.0

			-pregnancy and lactation				
Zhai et al., [12]	108 vs 76 controls	- AIS diagnosis<br><br>- admission within 72 h of onset	- hemorrhagic stroke,<br>-malignancy,<br>- severe liver and kidney dysfunctions<br>- history of cerebral hemorrhage or head trauma surgery.<br>- acute or chronic infection or trauma<br>- cerebral infarction or myocardial infarction within 3 months<br>- hormone and anti-inflammatory drug use within 3 months<br>- difficult cooperation, cognitive and communication difficulties<br>- surgery or lost to follow-up no	53.78±11.32 vs 54.07±11.18; M:F= 67: 39	Family history of stroke, Diabetes, Hypertension	- NIHSS score in the patient group (P<0.05),<br>- MoCA score lower in patient group (P<0.05)	NA
Ma et al., 2019 [13]	33 AIS + 20 healthy controls	- first ischemic stroke<br>- admission within 6 h of onset<br>- NIHSS<25<br>- CT/MRI showing focal neurological deficits	- recurrent stroke<br>- hematological diseases, malignancy, renal or liver failure<br>- mental disorders, severe dementia, or coronary artery disease<br>- other diseases affecting the hemogram	NA	No significant differences	NIHSS, BI, mRS	NA
Lin et al., 2022 [14]	96 AIS-59 FSTP & 37 NFSTP	- NIHSS ≥ 4<br>- diagnosis (clinical and CT/CTP) within ≤ 4 h of onset	- contraindications to thrombolysis<br>- other associated disorders, brain trauma and brain tumor	63.37 ± 7.09	No significant differences	NIHSS, mRS at 3 months	NIHSS-15.02 ± 5.27

		- occlusion of MCA M1, M2 / ICA on CTA	- anticoagulant treatment, INR >1.5,<br>- no CT examination or poor image quality<br>- no significant artery occlusion<br>- mental disorders or other diseases that affect examination				
Xiang et al., 2017 [15]	86 (40 AIS+46 controls)	AIS diagnosis (clinical+CT)<br>age>18,<br>aspirin upon admission,<br>NIHSS≥ 4,	Infection, severe liver, kidneyor cardiopulmonary disease, malignancy, rheumatic<br>immune disease, TIA, ICH,<br>previous AIS within last 3 months	Stroke: 63.1±11.7<br>Controls 65.5±11.4 controls;<br>Stroke: 63.0%<br>Controls: 61.5%	NR	NIHSS	12.0±6.2
Wang et al. 2020 [16]	76 (36 PSCI + 38 no PSCI)	history of a single stroke episode within the last year or compatible imaging imaging, cognitive impairment judged of vascular cause, conscious and stable,	other causes of cognitive impairment, long duration of cognitive impairment, treatment for cognitive impairment<br>, Beck Depression Inventory Score >13, alcohol or drug abuse, organ failure, pregnancy, lactation	PSCI: 67 (57–72)<br>PSCN: 68 (52–66)<br>(median and range);<br>PSCI: 44.4%,<br>No PSCI: 39.5%	No significant differences	MoCA<26 for PSCI,>=26 for PSCN	PSCI: 19 ±2.6 PSCN: 27±1.5
Jickling et al. 2016 [17]	212 (106 IS+106 controls)	AIS diagnosis (clinical+DWI-MRI)	Active or recent infection,<br>immunosuppression, leukemia, lymphoma	Stroke: 61.9±12.5Controls: 60±13.8;<br>Stroke: 51.8%<br>Controls: 49.1%	No significant differences	NIHSS	5.3 (2–9)

Wang et al. 2019 [18]	80 (40 IS+40 controls)	AIS diagnosis	Malignancy, autoimmune disease, "another unidentified disease", short survival time	Stroke:63 (50–75)<br>Controls: 63 (50–75) (median and range);<br>Stroke: 55%<br>Controls: 52.5%	BDNF	NIHSS	10 (5–15) (median and IQR)
Yuan et al. 2022 [19]	77 (45 PSCI, 32 PSCN)	AIS diagnosis (clinical + CT/MRI), age>18,<br>First AIS episode	Infection, other neurological disease, cancer, pregnancy	PSCI: 65.80±11.39<br>PSCN: 66.28±13.97;<br>PSCI:51.11%<br>PSCN:56.25%	Body weight	MMSE≤24 for PSCI, MMSE>24 for PSCN<br>mRS at diagnosis	PSCI mRS 3.53±1.10<br>PSCN mRS 3.19±0.97
Zhou and Qi 2021[20]	216 (108 IS+108 controls)	First AIS episode, diagnosis within 24 h	malignancy, severe liver and kidney disease, ICH	Stroke: 66.3±11.5<br>Controls: 64.5±12.8;<br>Stroke: 51.85%<br>Controls: 57.41%	BMI, total cholesterol, LDL, TG, Homocysteine	GOS at 30 days	NR
Ji et al. 2016 [21]	131 (65 IS+66 controls)	AIS diagnosis (clinical + CT/MRI)	ICH, unknown stroke etiology	Stroke: 64 (54–70)<br>Controls: 60 (53–64) (median and range);<br>Stroke: 61.5%<br>Controls: 54.5%	No significant differences	NIHSS	8 (6-11) (median and IQR)
He et al. 2019 [22]	94 (no controls)	AIS patients receiving rt-PA within 4.5 h of onset	Infection, severe kidney, liver, thyroid failure, rheumatic, or immune disease, hematologic disease and cancer, significant missing data.	68.3±12.6;<br>70.2%	No significant differences	NIHSS at diagnosis<br>mRS at 3 months	NIHSS:<br>5 (2-12) (median and IQR)

He et al. 2019 [23]	84 (no controls)	AIS patients receiving rt-PA within 4.5 h of onset	Infection, severe kidney, liver, thyroid failure, rheumatic, or immune disease, hematologic disease and cancer,	68.1 ± 12.2 70.2%	Smoking, Lipid-lowering medication use	NIHSS at diagnosis mRS at 3 months	NIHSS: 5 (2-12) (median and IQR)
Qi et al. 2021 [24]	20 (10 IS+10 controls)	AIS patients receiving rt-PA within 6 h of onset	NR	Stroke (grouped by serum collection timing): • 2h:72.5 ± 7.2 • 4h:73.0 ± 6.8 • 6h: 75.6 ± 7.2 Controls: 65.2 ± 8.0;	NR	NIHSS	NR
Jin and Xing 2017 [25]	216 (106 IS +110 controls)	AIS diagnosis (clinical + CT/MRI/MRA)	Infection, kidney or liver failure, malignancy, immunosuppression, thrombolysis	Stroke: 60.8 ± 9.7 Controls: 58.6 ± 15.2; Stroke: 45% Controls: 54%	No significant differences	NIHSS	NR
Jin and Xing 2018 [26]	296 (148 IS +148 controls)	AIS diagnosis (clinical + CT/MRI/MRA), age>18	Infection, kidney or liver failure, malignancy, immunosuppression, thrombolysis	Stroke: 61.5 ± 9.2 Controls: 59.8 ± 9.6; Stroke: 62.2% Controls: 56.8%	No significant differences	NIHSS	NR
Chen et al. 2020 [27]	430 (215 IS +215 controls)	age>18 Stroke: AIS diagnosis (clinical + CT/MRI) within 24 h of onset, Controls: at least 2 AIS risk factors	Severe infection, cerebral hemorrhage, epilepsy, malignancy, immunosuppression (<3 months), pregnancy, lactation	Stroke: 62.7 ± 11.0 Controls: 61.7 ± 9.3; Stroke: 72.6% Controls: 76.7%	Hypertension, diabetes mellitus, CKD	NIHSS	8.2 ± 3.5
Yang et al. 2018 [28]	141 (96 IS + 45 controls)	Stroke: AIS diagnosis (clinical + imaging) Controls: trauma and comorbidities, but	NR	Stroke: 61.5 (53–71) (median and IQR) Controls: NR;	NR	NIHSS	6 (2–11) (median and IQR)

		neurologically healthy		Stroke: 62.5%<br>Controls: NR			
Zhai et al. 2020 [12]	184 (108 IS+76 controls)	AIS diagnosis, diagnosis within 72 h of onset;	infection, hemorrhagic stroke, malignancy, history of brain hemorrhage or head trauma surgery, cerebral or myocardial infarction in the last 3 months<br>no hormones, no anti-inflammatory drugs (<3 months), no cognitive/communication barrier	Stroke: 53.78±11.32<br>Controls: 54.07±11.18;<br>Stroke: 62.04%<br>Controls: 51.32%	Family history of stroke, diabetes, hypertension	NIHSS and MoCA	NR
Liu et al. 2021 [29]	270 (170 IS +100 controls)	age>18<br>Stroke: AIS diagnosis (ASA criteria),<br>no cerebral hemorrhagic infarction<br>Controls: at least 2 risk factors for AIS	Infection, malignancy, chronic inflammatory/immune diseases, pregnancy, lactation	Stroke: 66.7±9.8<br>Controls: 65.3±8.4;<br>Stroke: 70%<br>Controls: 66%	Hyperuricemia	NIHSS	10 (6–12) (median and IQR)
Zhou and Zhang 2014 [30]	89 (68 IS+21controls)	Stroke: AIS diagnosis (clinical + MRI/MRA)<br>Controls: age-matched	Acute infection, malignancy, liver or kidney failure, immune diseases	Stroke: 66 (55-76)<br>Controls: 58 (54-67) (median and range);<br>Stroke: 66.2%<br>Controls: 47.6%	NR	NIHSS	NR
Wang et al. 2015 [31]	117 (58 IS+59 controls)	Stroke: AIS diagnosis (clinical + imaging)<br>Controls: age and gender-matched	Acute infection, history of stroke, intracranial tumor, polytrauma, hematological diseases, kidney or liver failure, “other diseases	Stroke: 61.7±6.3<br>Controls: 63.5±9.6;<br>Stroke: 75.9%<br>Controls: 69.5%	Hypertension, diabetes, and hyperlipidemia	NIHSS at diagnosis<br>mRS at 3 months (<2: good outcome)	4.22 (0-12) (mean and range)

			affecting the hemogram"				
Ma et al. 2020 [32]	100 (60 IS+40 controls)	AIS diagnosis	NR	NR;<br>NR	No significant differences	NIHSS	NR
Abdelaleem et al. 2022 [33]	223 (77 diabetics with stroke, 75 diabetics without stroke, 71 controls)	AIS diagnosis (clinical + CT/MRI)	Acute infection, cerebral hemorrhage, recurrent stroke, family history of stroke, head trauma, hypertension, liver or kidney disease, immune diseases, hematological diseases	Diabetics with stroke: 57.08 ± 16.31<br>Diabetics without stroke: 53.19 ± 17.78<br>Controls:54.58 ± 18.75;<br>Diabetics with stroke: 61.0%<br>Diabetics without stroke:65.3%<br>Controls: 64.8%	Diabetics with stroke vs diabetics without stroke: BMI, fasting blood glucose, 2 h post-prandial glucose, LDL (higher for diabetics with stroke)	NIHSS	11.38 ± 5.12
Xue et al. 2018 [34]	125 (65 IS+55Controls)	AIS diagnosis (clinical + CT/MRI) diagnosis within 24 h of onset	NR	Stroke: 65.2±6.7<br>Controls: 62.3±7.4;<br>Stroke: 61.5%<br>Controls: 50.9%	No significant differences	NIHSS	NR
Xie et al. 2019 [35]	80<br>(40 IS+40 Controls)	AIS diagnosis (clinical + MRI), infarction volume assessed by MRI	NR	NR	NR	NIHSS	NR
Guo et al. 2020 [36]	235<br>(170 IS +65 Controls)	NA	NA	NA	NA	NIHSS	NA
Yuan et al., 2016 [37,38]	288 (152 IS+ 136 Controls)	AIS diagnosis (clinical + MRI/MRA) within 24 h of onset	- acute infection<br>- liver, kidney disease<br>- autoimmune disease<br>- malignancy<br>- pregnancy,	63.8 ± 11.4 vs 61.7 ± 11.8<br>M:F= 100/52 vs 88/48	Smoking, hypertension, diabetes, dyslipidemia (LDL, HDL)	NIHSS	Patients with high scores (0.48 ± 0.25) and patients with low

		- large artery atherosclerotic cerebral infarction<br>- Hospital stay ≤ 14 days.					scores (0.74 ± 0.32)
Zhaou et al., 2016 [39]	272 (168 IS + 104 Controls)	AIS diagnosis (clinical+ MRI/MRA) within 24 h of onset	recurrent stroke, blood disorders, acute infectious diseases, renal or liver failure, malignancy	70 ± 8 vs 69 ± 9<br>M:F= 88/80	No significant differences	NIHSS	NR
Zhong et al., 2021 [40]	128 (89 IS + 39 Controls)	AIS patients	- infectious disease or antibiotics within 3 months<br>- other cardiovascular and cerebrovascular diseases<br>- autoimmune disease<br>- psychiatric disorders<br>- malignancy<br>- organ dysfunction or treatment<br>- surgery within 3 months<br>- pregnancy, lactation<br>- life expectancy <1 month	59.96 ± 7.24 vs 59.21 ± 7.18<br>M:F=46:43	NR	NIHSS	20.39 ± 7.43
Song et al., 2021 [41]	110 (80 IS + 30 Controls)	AIS diagnosis (clinical + MRI)	- patients not receiving vasodilation or thrombolysis before blood sampling<br>- head trauma or surgery<br>- cancer, immune disorders	57.0 ± 21.1 vs 6.0 ± 20.9<br>M:F= 53/27	Diabetes mellitus, Hyperlipidemia	NIHSS	7.89 ± 1.42
Zhang et al., 2020 [42]	163 (93 IS+70 Controls)	-age 18–85<br>- AIS diagnosis (clinical + CT/ MRI)	-Patients received rt-PA or thrombectomy	67.5 ± 11.2 vs 66.2 ± 10.8<br>M:F=57/36	systolic blood pressure, glucose, lipoprotein	NIHSS	

			-ICH or other intracranial pathology (vascular malformation, tumor, abscess, other nonischemic disease),<br>- prior stroke, myocardial infarction, atrial fibrillation, congestive heart failure<br>-pregnancy, lactation		phospholipase A, intima-media thickness, homocysteine		
Zeng et al, 2013 [43]	105 (40 good outcomes + 65 poor outcomes)	AIS diagnosis (clinical + imaging)	prior stroke<br>abnormal liver, kidney function<br>- infectious diseases<br>- immune diseases<br>- malignancy	66 (54-74) for good outcome group vs 68 (57-78) for poor outcome group	IL-6, FDP, PAI-1, differ between stroke patients with mRS≤2 vs mRS>2	NIHSS, mRS	
Yang et al., 2020 [44]	139 (79 AIS vs 60 healthy controls)	- AIS diagnostic (clinical + CT/MRI)	- hemorrhagic stroke, brain trauma, hypertensive encephalopathy, encephalitis<br>- history of stroke or head trauma surgery<br>-other neurological diseases (meningitis, myelitis, epilepsy, multiple sclerosis)<br>- brain malignancy;	54.68±10.98 vs 53.72±11.26<br>M:F=55/21	Hypertension, Hemocysteine level, Uric acid, FBG, Platelets, INR	NIHSS	18.82±5.14
Liu et al., 2019 [45]	65 (40 AIS + 25 healthy controls)	- first ischemic<br>- AIS diagnosis (clinical + MRI)<br>- age 55–65<br>- admission within 72 h of onset;<br>-NIHSS 4–15;<br>- large-artery atherosclerosis.	NR	55–65	No significant differences	NIHSS<br>mRS	

Zhao et al., 2020 [46]	69 (43 AIS + 26 healthy controls)	- AIS diagnosis (clinical + MRI)	- cerebral infarction secondary to other conditions<br>- diabetes mellitus, coronary artery disease hypertension, kidney disease, hematological and autoimmune diseases	60.41 ± 10.11 vs 57.96 ± 10.13, M:F= 54/22	No significant differences	NIHSS	
Sheikhbahaei et al., 2019 [47]	50 (33 AIS vs 17 controls)	- AIS diagnosis (clinical + CT/MRI) within 72 h of onset	- history of GI and CNS tumors, - pulmonary, neurodegenerative, cardiovascular, autoimmune diseases<br>- prior stroke (<1 year),<br>- hemorrhagic transformation or AMI during hospital stay	67.9 ± 11.3 AIS vs 66.85 ± 11 controls<br><br>M:F=20/13	NR	NIHSS<br><br>mRS acute<br><br>mRS chronic	12 ± 5.25<br><br>3.35 ± 1.25<br><br>2.6 ± 3.4
Guo et al., 2022 [48]	192 (142 AIS + 50 controls)	- AIS diagnosis according to Chinese Stroke Association criteria and CT/MRI<br>- admission within 24 h of onset	- hemorrhagic stroke, traumatic brain injury, hypertensive encephalopathy, encephalitis, other cerebrovascular diseases.<br>- cardiopulmonary disease, multiple sclerosis, malignancy, hematological disease<br>-altered state of consciousness, psychiatric disorders	52.40±2.35 vs 52.15±2.83<br><br>M:F=97:45	No significant differencesc	NIHSS<br><br><br><br><br>mRS	10.50±3.25 (good prognosis group)<br><br>17.62±3.58 (poor prognosis group)<br><br>1.42±0.28 (good prognosis group)<br><br>4.17±0.56 (poor prognosis group)

Fu et al., 2019 [49]	205 (108 AIS + 97 controls)	- AIS diagnosis (CT/MRI)	- thrombolytic therapy, renal or liver failure, tumor, infectious disease, hematologic disease.	64 ± 9.2 vs 59 ± 6.4<br><br>M:F=75/33	Diabetes, Cardiopathy	NIHSS	3.86 (1, 12)
Ye et al., 2021 [50]	86 (43 AIS vs 43 controls)	- AIS diagnosis (MRI)	NR	64.42 ± 10.25 vs 63.33 ± 14.32<br><br>M:F=22/21	No significant differences	NIHSS<br><br>mRS	3.30 ± 1.67<br><br>mRS ≤ 2 28 (64.0%)<br>mRS > 2 15(36.0%)
Otero-Ortega et al., 2021 [51]	103 (81 AIS vs 22 controls)	- age>18<br>- admission within 25 h of onset<br>- pre-stroke mRS ≤1<br>- SC or CSC lesion and compatible clinical presentation	-prior ischemic stroke, dementia, transient ischemic attack, brain tumor, cerebral hemorrhage,<br>- drug or alcohol abuse<br>- clinical conditions that precluded diagnosis<br>- participation in a clinical trial	CSC: 70.65 (15.39), SC:61.65 (11.83) vs controls 61(12.74)	Hypertension, Dyslipidemia, Smoker, Atrial fibrillation, Charlson comorbidity index,	NIHSS at admission<br><br>NIHSS at 3 months<br><br>mRS at 3 months	CSC: 9.00 (9.75) and SC: 3.00 (3.25)<br><br>CSC: 0.00 (1.00) and SC: 0.00 (1.00)<br><br>CSC: 1.00 (2.00) and SC: 1.00 (2.00)
Yang et al., 2016 [52]	172 (114 AIS vs 58 controls)	- AIS diagnosis (clinical + CT/MRI)	- embolic brain infarction, transient ischemic attack, cerebrovascular malformation, subarachnoid hemorrhage, brain tumor<br>- severe systemic diseases (collagenosis, liver or renal)	61±11.3 vs 56±3.9<br>M:F=78 (68.4)	Cholesterol, mmol/L, HDL-C, mmol/L, Hypertension, Diabetes mellitus, Smoking	NIHSS	

Zhou et al., 2018 [53]	100 (50 AIS vs 50 controls)	- AIS diagnosis (MRI)	NR	65.42 ± 10.25 vs 63.33 ± 14.32<br>M:F=26/24	Diabetes mellitus, Hyperlipidemia	NIHSS score<br><br>mRS ≤2<br><br>mRS>2	7.82 ± 1.36<br><br>32 (64.0%)<br><br>18 (36.0%)
Chen et al., 2018 [54]	230 (128 AIS vs 102 controls)	- admission within 24 hours of onset<br>- age >18	- suspected infection<br>-thrombolytic or anticoagulant therapies<br>- ICH, transformation, other neurological complications,<br>- malignancy, - comorbidities, proinflammatory conditions	68.42±17.26 vs 65.36±16.32<br>M:F=109 (85.2%)	No significant differences	NIHSS	6.2 (±7.4)
Chen et al., 2017 [55]	83 (50 AIS + 33 controls)	- AIS diagnosis	- acute infectious disease<br>-recurrent stroke<br>- presentation within more than 72 h of onset<br>- renal or liver failure<br>- malignancy<br>- hematological disease<br>- difficult cooperation	64 ± 9.4 vs 63 ± 7.9<br>M:F=32/18	Diabetes mellitus, Cardiopathy	NIHSS<br><br>mRS≤2<br><br>mRS>2	3.32 (0.14)<br><br>30 (60.0%)<br><br>20 (40.0%)
Liang et al., 2016 [56]	199 (102 AIS + 97 controls)	-AIS diagnosis (clinical + CT/MRI)	NR	65.1±10.0<br>vs<br>6.52 (51~68)<br>M:F= 69/33	Hypertension, Diabetes, Hyperlipidemia, Cardiopathy	NIHSS	3.95 (0, 20)
Wang et al., 2014 [57]	154 (79 AIS + 75 controls)	-AIS diagnosis (CT/MRI/MRA)<br>-<72 h from onset to sample collection	- Acute infectious disease<br>- recurrent stroke, intracranial tumor, multiple trauma<br>- liver, kidney failure<br>- hematological disease, other diseases	65.1 ± 10.0 vs 62.5 ± 6.3<br>M:F=58/21	Hypertension, Diabetes, Hyperlipidemia	NIHSS	3.95 (0, 20)

			affecting the hemogram				
Wang et al., 2021 [58]	176 (88 AIS vs 88 controls)	- AIS diagnosis (clinical + CT /MRI)<br>- diagnosis within 6 h of onset<br>- age > 18	- history of ICH or intracranial tumor<br>- heart, liver and kidney failure,	63.22±5.43 vs 62.42±3.64<br><br>M:F= 48/40	-BMI<br>Hypertension<br>Hyperlipidemia (HDL, LDL)<br>Diabetes<br>-	mRS	-
Jia et al., 2015 [59]	242 (146 AIS vs 96 controls)	Admission within 24 h of<br>- age > 18	- suspected infection<br>-thrombolytic or anticoagulant therapies<br>- ICH, , transformation, other neurological complications,<br>- malignancy, - comorbidities, proinflammatory conditions	67.29 ± 14.16 vs 63.23 ± 15.24<br>Stroke: 112 (76.7%)	-	NIHSS	5.3 (±8.2)
Liang et al., 2019 [60]	124 (62 AIS + 62 controls)	- AIS diagnosis (clinical + CT/MRI) - Age > 18;<br>- admission within 7 days of s onset<br>- anticipated life expectancy of at least 6 months.	- history of major depressive disorder or other psychiatric disorders, family history of depression<br>- altered state of consciousness	Early onset PSD: 68.02 ± 9.86 vs Late-onset PSD: 66.89 ± 7.36<br>Controls: 65.56 ± 11.95<br>M:F=40:22	Hypertension, Carotid Artery Stenosis, Fasting blood-glucose, HbA1c,	NIHSS	Early onset PSD: 4.86 ± 3.19<br><br>Late-onset PSD:4.21 ± 2.35
			- difficult cooperation due to aphasia, hearing impairment, severe comprehension or cognitive deficit<br>- other life-threatening or severe illness			HAMD 2–3 weeks	Early onset PSD: 9 [8,15]<br><br>Late-onset PSD: 4 [3,4]
						HAMD 3 months	Early onset PSD: 8 [7,11]

							Late-onset PSD: 9 [9,10]
Hu et. al, 2020 [61]	257 (73 PSD group, 184 non-PSD group)	-AIS diagnosis byWHO-MONICA criteria,<br>- MRI assessment within 24 h of admission;<br>- < 1 week from onset to diagnosis;<br>- age > 18 years.	- psychiatric conditions s significant cognitive impairment, severe aphasia or dysarthria, visual or auditory impairment<br>- metabolic abnormalities<br>- tumors<br>- other medical illness<br>- patients without MRI scans	PSD group: 62.7±10.1 vs Non-PSD group: 62.1±10.3<br>M:F= 151:72	BMI	NIHSS<br><br>HAMD	PSD group: 9 (7-12) vs Non-PSD group: 8 (6-11)
Cui et al., 2021 [62]	136 (76 PSD + 60 Non-PSD)	-initially confirmed with stroke;	- acute infectious disease<br>- other brain diseases<br>- malignancy<br><br>- hormone and anti-inflammatory use in the past 3 months<br>-cognitive impairment	<45: PSD: 40 (52.63)<br>≥45: PSD: 36 (47.37)<br><45: Non-PSD: 28 (46.67) ≥45: Non-PSD: M:F=32 (53.33) M:F=48:28	History of mental illness	NIHSS<br><br>HAMD	4.84±2.30<br><br>6.32±2.03
Zeng et al., 2011 [63]	172 (112 AIS+ 60 controls)	AIS diagnosis (clinical + DWI-MRI and MRA)	- prior stroke<br>- acute infectious disease<br>- malignancy<br>- immune, hematological disease<br>- liver, kidney failure	AIS: 68(57,76) vs 67(64,73)<br>M:F= 78/34	No significant differences	NIHSS	5 (1,10)

Notes: Values denoted as *a±b* represent mean and standard deviation, those denoted as *a (b)* denote median and interquartile range, those denoted as a (b-c) denote median and range. Significant differences for age and gender are marked. Abbreviations AIS, Acute ischemic stroke; ASA, American Stroke Association; AST, Aspartate aminotransferase; BDI, Beck Depression Inventory; BDNF, Brain derived neurotrophic factor; BI, Barthel Index; BI, Body Index; BMI, Body mass index; BT, Body temperature; CKD, Chronic kidney disease; CSC, Cortical-subcortical; CT, Computed tomography; CRP, C Reactive Protein; CTP, CT perfusion; DWI-MRI, Diffusion-weighted imaging-MRI; EMVs, Endothelial Microvesicles; FBG, fasting blood glucose; FDP, Fibrin degradation product; FSTP, Favorable short-term prognosis; GCS, Glasgow Coma Scale; GOS, Glasgow Outcome Scale; HbA1C, Glucose-bound (glycated) hemoglobin; HDL-C, HAMD, Hamilton depression scale; High-density

lipoprotein-cholesterol; ICH, intracerebral hemorrhage; IL-6, Interleukin-6; INR, International normalized ratio; LAA, Large-artery atherosclerosis; LDL-C, low-density lipoprotein cholesterol; MCA, Middle Cerebral Artery; MMSE, Mini-Mental State Examination; MoCA, Montreal Cognitive Assessment scale; miR, microRNAs; MRA, Magnetic Resonance Angiography; MRI: Magnetic resonance imaging; mRS, Modified Rankin score; NA, not applicable; NIHSS, National Institutes of Health Stroke Scale; NFSTP, Unfavorable short-term prognosis; NR, not reported; PAI-1, Plasminogen Activator Inhibitor 1; PSCI, post-stroke cognitive impairment; PSCN, post-stroke cognitive normality; PSD, Post-stroke depression; rt-PA, Recombinant tissue plasminogen activator; SAO, Small artery occlusion; SC, Subcortical; TG, Triglycerides; TIA, Transient ischemic attack; WBC, White blood cells;

Supplemental references

1. Ma X, Liao X, Liu J, Wang Y, Wang X, Chen Y, et al. Circulating endothelial microvesicles and their carried miR-125a-5p: potential biomarkers for ischaemic stroke. Stroke Vasc Neurol [Internet]. BMJ Specialist Journals;

2. Peng G, Yuan Y, Wu S, He F, Hu Y, Luo B. MicroRNA let-7e Is a Potential Circulating Biomarker of Acute Stage Ischemic Stroke. Transl Stroke Res. 2015;6:437–45.

3. Wu J, Fan C-L, Ma L-J, Liu T, Wang C, Song J-X, et al. Distinctive expression signatures of serum microRNAs in ischaemic stroke and transient ischaemic attack patients. Thromb Haemost. 2017;117:992–1001.

4. Kijpaisalratana N, Nimsamer P, Khamwut A, Payungporn S, Pisitkun T, Chutinet A, et al. Serum miRNA125a-5p, miR-125b-5p, and miR-433-5p as biomarkers to differentiate between posterior circulation stroke and peripheral vertigo. BMC Neurology. 2020;20:372.

5. Kautzky V. Identifizierung von Einflussfaktoren auf die Konzentrationen der zirkulierenden microRNAs miR-125a-5p, miR-125b-5p und miR-143-3p nach ischämischem Schlaganfall [.PhDThesis]. Ludwig-Maximilians-Universität München; 2022.

6. Zhu X, Ding J, Wang B, Wang J, Xu M. Circular RNA DLGAP4 is down-regulated and negatively correlates with severity, inflammatory cytokine expression and pro-inflammatory gene miR-143 expression in acute ischemic stroke patients. Int J Clin Exp Pathol. 2019;12:941–8.

7. Niu M, Li H, Li X, Yan X, Ma A, Pan X, et al. Circulating Exosomal miRNAs as Novel Biomarkers Perform Superior Diagnostic Efficiency Compared With Plasma miRNAs for Large-Artery Atherosclerosis Stroke. Front Pharmacol. 2021;12:791644.

8. Li G, Ma X, Zhao H, Fan J, Liu T, Luo Y, et al. Long non-coding RNA H19 promotes leukocyte inflammation in ischemic stroke by targeting the miR-29b/C1QTNF6 axis. CNS Neuroscience & Therapeutics. 2022;28:953–63.

9. Kotb HG, Ibrahim AH, Mohamed EF, Ali OM, Hassanein N, Badawy D, et al. The expression of microRNA 146a in patients with ischemic stroke: an observational study. Int J Gen Med. 2019;12:273–8.

10. Wu X, Zhang X, Li D, Zhu Z. Plasma level of miR-99b may serve as potential diagnostic and short-term prognostic markers in patients with acute cerebral infarction. J Clin Lab Anal. 2020;34:e23093.

11. Huang S, Zhao J, Huang D, Zhuo L, Liao S, Jiang Z. Serum miR-132 is a risk marker of post-stroke cognitive impairment. Neurosci Lett. 2016;615:102–6.

12. Zhai Y, Zhu Z, Li H, Zhao C, Huang Y, Wang P. miR-195 and miR-497 in acute stroke and their correlations with post-stroke cognitive impairment. Int J Clin Exp Pathol. 2020;13:3092–9.

13. Ma Q, Li G, Tao Z, Wang J, Wang R, Liu P, et al. Blood microRNA-93 as an indicator for diagnosis and prediction of functional recovery of acute stroke patients. J Clin Neurosci. 2019;62:121–7.

14. Lin X, Wang W, Tao T, Zhang D, Mao L, He X. Synthetic role of miR-411-5p and CT perfusion information in predicting clinical outcomes after thrombolysis in acute cerebral infarction. Acta Neurol Belg. 2022;

15. Xiang W, Tian C, Lin J, Wu X, Pang G, Zhou L, et al. Plasma let-7i and miR-15a expression are associated with the effect of recombinant tissue plasminogen activator treatment in acute ischemic stroke patients. Thromb Res. 2017;158:121–5.

16. Wang Z-Q, Li K, Huang J, Huo T-T, Lv P-Y. MicroRNA Let-7i Is a Promising Serum Biomarker for Post-stroke Cognitive Impairment and Alleviated OGD-Induced Cell Damage in vitro by Regulating Bcl-2. Front Neurosci. 2020;14:215.

17. Jickling GC, Ander BP, Shroff N, Orantia M, Stamova B, Dykstra-Aiello C, et al. Leukocyte response is regulated by microRNA let7i in patients with acute ischemic stroke. Neurology. 2016;87:2198–205.

18. Wang J, Huang Q, Ding J, Wang X. Elevated serum levels of brain-derived neurotrophic factor and miR-124 in acute ischemic stroke patients and the molecular mechanism. 3 Biotech. 2019;9:386.

19. Yuan M, Guo Y-S, Zhang X-X, Gao Z-K, Shen X-Y, Han Y, et al. Diagnostic performance of miR-21, miR-124, miR-132, and miR-200b serums in post-stroke cognitive impairment patients. Folia Neuropathol. 2022;60:228–36.

20. Zhou X, Qi L. miR-124 Is Downregulated in Serum of Acute Cerebral Infarct Patients and Shows Diagnostic and Prognostic Value. Clin Appl Thromb Hemost. 2021;27:10760296211035446.

21. Ji Q, Ji Y, Peng J, Zhou X, Chen X, Zhao H, et al. Increased Brain-Specific MiR-9 and MiR-124 in the Serum Exosomes of Acute Ischemic Stroke Patients. PLoS One. 2016;11:e0163645.

22. He X-W, Shi Y-H, Liu Y-S, Li G-F, Zhao R, Hu Y, et al. Increased plasma levels of miR-124-3p, miR-125b-5p and miR-192-5p are associated with outcomes in acute ischaemic stroke patients receiving thrombolysis. Atherosclerosis. 2019;289:36–43.

23. He X-W, Shi Y-H, Zhao R, Liu Y-S, Li G-F, Hu Y, et al. Plasma Levels of miR-125b-5p and miR-206 in Acute Ischemic Stroke Patients After Recanalization Treatment: A Prospective Observational Study. J Stroke Cerebrovasc Dis. 2019;28:1654–61.

24. Qi Z, Zhao Y, Su Y, Cao B, Yang J-J, Xing Q. Serum Extracellular Vesicle–Derived miR-124-3p as a Diagnostic and Predictive Marker for Early-Stage Acute Ischemic Stroke. Frontiers in Molecular Biosciences. 2021;8.

25. Jin F, Xing J. Circulating pro-angiogenic and anti-angiogenic microRNA expressions in patients with acute ischemic stroke and their association with disease severity. Neurol Sci. 2017;38:2015–23.

26. Jin F, Xing J. Circulating miR-126 and miR-130a levels correlate with lower disease risk, disease severity, and reduced inflammatory cytokine levels in acute ischemic stroke patients. Neurol Sci. 2018;39:1757–65.

27. Chen X, Zhang X, Su C, Huang S. Long noncoding RNA HULC in acute ischemic stroke: Association with disease risk, severity, and recurrence-free survival and relation with IL-6, ICAM1, miR-9, and miR-195. J Clin Lab Anal. 2020;34:e23500.

28. Yang G, Liu Z, Wang L, Chen X, Wang X, Dong Q, et al. MicroRNA-195 protection against focal cerebral ischemia by targeting CX3CR1. J Neurosurg. 2018;1–10.

29. Liu C, Huang H, Li Y, Zhao H. The relationship of long non-coding RNA maternally expressed gene 3 with microRNA-21 and their correlation with acute ischemic stroke risk, disease severity and recurrence risk. Clin Neurol Neurosurg. 2021;210:106940.

30. Zhou J, Zhang J. Identification of miRNA-21 and miRNA-24 in plasma as potential early stage markers of acute cerebral infarction. Mol Med Rep. 2014;10:971–6.

31. Wang Y, Huang J, Ma Y, Tang G, Liu Y, Chen X, et al. MicroRNA-29b is a therapeutic target in cerebral ischemia associated with aquaporin 4. J Cereb Blood Flow Metab. 2015;35:1977–84.

32. Ma X, Yun HJ, Elkin K, Guo Y, Ding Y, Li G. MicroRNA-29b Suppresses Inflammation and Protects Blood-Brain Barrier Integrity in Ischemic Stroke. Mediators of Inflammation. Hindawi; 2022;2022:e1755416.

33. Abdelaleem OO, Shaker OG, Mohamed MM, Ahmed TI, Elkhateeb AF, Abdelghaffar NK, et al. Differential Expression of Serum TUG1, LINC00657, miR-9, and miR-106a in Diabetic Patients With and Without Ischemic Stroke. Frontiers in Molecular Biosciences. 2022;8.

34. Xue Y, Li M, Liu D, Zhu Q, Chen H. Expression of miR-9 in the serum of patients with acute ischemic stroke and its effect on neuronal damage. Int J Clin Exp Pathol. 2018;11:5885–92.

35. Xie Z, Liu B, Zhou M, Chen Y. Expression and clinical significance of plasma miR-124 in acute ischemic stroke. The Journal of Practical Medicine. 2019;343–5.

36. Guo C, Zhong C, Li Q, Gao Y, Li W, Ou Y. [Expressions and neural function prognostic evaluation of serum microRNA-24 and microRNA-29b in elderly patients with acute ischemic stroke]. Zhonghua Wei Zhong Bing Ji Jiu Yi Xue. 2020;32:78–82.

37. Yuan M, Yuan H, Zhou C, Liu F, Lin C, Tang Y. The significance of low plasma miR-335 level in patients with acute cerebral infarction may be associated with the loss of control of CALM1 expression. Int J Clin Exp Med. Madison: E-Century Publishing Corp; 2016;9:19595–601.

38. Yuan M, Tang Y, Zhou C, Liu F, Chen L, Yuan H. Elevated plasma CaM expression in patients with acute cerebral infarction predicts poor outcomes and is inversely associated with miR-26b expression. Int J Neurosci. 2016;126:408–14.

39. Zhao B, Zhu Z, Hao J, Wan Z, Guo X. Decreased plasma miR-335 expression in patients with acute ischemic stroke and its association with calmodulin expression. J Int Med Res. 2016;44:1331–8.

40. Zhong C, Yin C, Niu G, Ning L, Pan J. MicroRNA miR-497 is closely associated with poor prognosis in patients with cerebral ischemic stroke. Bioengineered. 2021;12:2851–62.

41. Song X-D, Li S-X, Zhu M. Plasma miR-409-3p promotes acute cerebral infarction via suppressing CTRP3. Kaohsiung J Med Sci. 2021;37:324–33.

42. Zhang H, Chen G, Qiu W, Pan Q, Chen Y, Chen Y, et al. Plasma endothelial microvesicles and their carrying miRNA-155 serve as biomarkers for ischemic stroke. J Neurosci Res. 2020;98:2290–301.

43. Zeng L, Liu J, Wang Y, Wang L, Weng S, Chen S, et al. Cocktail blood biomarkers: prediction of clinical outcomes in patients with acute ischemic stroke. Eur Neurol. 2013;69:68–75.

44. Yang S, Zhan X, He M, Wang J, Qiu X. miR-135b levels in the peripheral blood serve as a marker associated with acute ischemic stroke. Exp Ther Med. 2020;19:3551–8.

45. Liu P, Han Z, Ma Q, Liu T, Wang R, Tao Z, et al. Upregulation of MicroRNA-128 in the Peripheral Blood of Acute Ischemic Stroke Patients is Correlated with Stroke Severity Partially through Inhibition of Neuronal Cell Cycle Reentry. Cell Transplant. 2019;28:839–50.

46. Zhao H, Li G, Wang R, Tao Z, Ma Q, Zhang S, et al. Silencing of microRNA-494 inhibits the neurotoxic Th1 shift via regulating HDAC2-STAT4 cascade in ischaemic stroke. Br J Pharmacol. 2020;177:128–44.

47. Sheikhbahaei S, Manizheh D, Mohammad S, Hasan TM, Saman N, Laleh R, et al. Can MiR-503 be used as a marker in diabetic patients with ischemic stroke? BMC Endocr Disord. 2019;19:42.

48. Guo C, Yao Y, Li Q, Gao Y, Cao H. Expression and Clinical Value of miR-185 and miR-424 in Patients with Acute Ischemic Stroke. Int J Gen Med. 2022;15:71–8.

49. Fu C, Chen S, Cai N, Liu Z, Wang P, Zhao J. Potential Neuroprotective Effect of miR-451 Against Cerebral Ischemia/Reperfusion Injury in Stroke Patients and a Mouse Model. World Neurosurg. 2019;130:e54–61.

50. Ye Z, Hu J, Xu H, Sun B, Jin Y, Zhang Y, et al. Serum Exosomal microRNA-27-3p Aggravates Cerebral Injury and Inflammation in Patients with Acute Cerebral Infarction by Targeting PPARγ. Inflammation. 2021;44:1035–48.

51. Otero-Ortega L, Alonso-López E, Pérez-Mato M, Laso-García F, Gómez-de Frutos MC, Diekhorst L, et al. Circulating Extracellular Vesicle Proteins and MicroRNA Profiles in Subcortical and Cortical-Subcortical Ischaemic Stroke. Biomedicines. 2021;9:786.

52. Yang Z-B, Li T-B, Zhang Z, Ren K-D, Zheng Z-F, Peng J, et al. The Diagnostic Value of Circulating Brain-specific MicroRNAs for Ischemic Stroke. Intern Med. 2016;55:1279–86.

53. Zhou J, Chen L, Chen B, Huang S, Zeng C, Wu H, et al. Increased serum exosomal miR-134 expression in the acute ischemic stroke patients. BMC Neurol. 2018;18:198.

54. Chen Z, Wang K, Huang J, Zheng G, Lv Y, Luo N, et al. Upregulated Serum MiR-146b Serves as a Biomarker for Acute Ischemic Stroke. Cell Physiol Biochem. 2018;45:397–405.

55. Chen Y, Song Y, Huang J, Qu M, Zhang Y, Geng J, et al. Increased Circulating Exosomal miRNA-223 Is Associated with Acute Ischemic Stroke. Front Neurol. 2017;8:57.

56. Liang T, Lou J. Increased Expression of mir-34a-5p and Clinical Association in Acute Ischemic Stroke Patients and in a Rat Model. Med Sci Monit. 2016;22:2950–5.

57. Wang Y, Zhang Y, Huang J, Chen X, Gu X, Wang Y, et al. Increase of circulating miR-223 and insulin-like growth factor-1 is associated with the pathogenesis of acute ischemic stroke in patients. BMC Neurol. 2014;14:77.

58. Wang Q, Wang F, Fu F, Liu J, Sun W, Chen Y. Diagnostic and prognostic value of serum miR-9-5p and miR-128-3p levels in early-stage acute ischemic stroke. Clinics (Sao Paulo). 2021;76:e2958.

59. Jia L, Hao F, Wang W, Qu Y. Circulating miR-145 is associated with plasma high-sensitivity C-reactive protein in acute ischemic stroke patients. Cell Biochemistry and Function. 2015;33:314–9.

60. Liang H-B, He J-R, Tu X-Q, Ding K-Q, Yang G-Y, Zhang Y, et al. MicroRNA-140-5p: A novel circulating biomarker for early warning of late-onset post-stroke depression. J Psychiatr Res. 2019;115:129–41.

61. Hu J, Zhou W, Zhou Z, Yang Q, Xu J, Dong W. miR-22 and cerebral microbleeds in brainstem and deep area are associated with depression one month after ischemic stroke. Braz J Med Biol Res. 2020;53:e9162.

62. Cui Y, Ma G, Kong F, Song L. Diagnostic Values of miR-221-3p in Serum and Cerebrospinal Fluid for Post-Stroke Depression and Analysis of Risk Factors. Iranian Journal of Public Health. Tehran University of Medical Sciences; 2021;50:1241.

63. Zeng L, Liu J, Wang Y, Wang L, Weng S, Tang Y, et al. MicroRNA-210 as a novel blood biomarker in acute cerebral ischemia. Front Biosci (Elite Ed). 2011;3:1265–72.
