# Supplementary material for: Circulating MicroRNAs and Extracellular Vesicle-Derived MicroRNAs as Predictors of Functional Recovery in Ischemic Stroke Patients: A Systematic Review and Meta-Analysis
Source: Int J Mol Sci. 2022 Dec 23;24(1):251. doi: 10.3390/ijms24010251 (PMC9820088; doi:10.3390/ijms24010251)
Supplement: Supplementary file 1 [file ijms-24-00251-s001.zip › Supplimental S2.pdf]

Table S2. Prognostic performance of biomarkers included

Biomarker	Sample size	First sample collection (after admission)	miRNA level of expression (up/downregulated)	Discrimination task — Score evaluated	Cut-off	AUC (95% CI)	Se (%)	Sp (%)	Ref
miR-132	76 IS patients (39 PSCI + 37 PSCN) vs 38 age-matched controls	Chronic (within 1 year after stroke onset)	Upregulated in PSCI vs PSCN	Distinguishing PSCI vs PSCN — MoCA	0.697	0.961 (0.931-0.991)	94.9	86.7	[1]
miR-411-5p	96 ACI patients	24 h after thrombolysis and 3 months (90 days) after onset	Increased at 24 h after thrombolysis and at 3 months after onset	FSTP vs NFSTP — NIHSS, mRS	>0.915	0.900 (NR)	86.44	86.49	[2]
let-7i	36 PSCI vs 38 PSCN	3 (1–5) days after cognitive symptoms	Upregulated in PSCI vs PSCN	Predicting PSCI — MoCA	NA	0.859 (0.777–0.941)	94.7	86.5	[3]
miR-124-3p	84 AIS (55 FSP vs 29 UFSP)	24 h after rtPA	Upregulated in unfavorable outcome patients	Predicting unfavorable outcome — NIHSS, mRS	NR	0.707 (0.594-0.804)	66.67	79.23	[4]
miR-125b-5p	84 AIS (55 FSP vs 29 UFSP)	24 h after rtPA	Higher expression 24h after thrombolysis		NR	0.729 (0.616-0.823)	92.86	46.00	
miR-192-5p	84 AIS (55 FSP vs 29 UFSP)	24 h after rtPA			NR	0.647 (0.533-0.750)	92.86	35.85	
miR-125b-5p	94 AIS (61 + 23)	24 h after rtPA	High expression in patients with a moderate-to-severe stroke	Predicting unfavorable outcome — NIHSS, mRS	1.184×10^{-3}	0.735 (0.623-0.829)	86.36	55.36	[5]

			Upregulated in unfavorable/poor outcome patients						
miR-29b	170 vs 65 healthy	Unclear	Downregulated in poor prognosis patients	Predicting poor neurological outcome — NIHSS	0.48	0.835, (0.778-0.890)			[6]
miR-24	170 vs 65 healthy	Unclear	Downregulated in AIS patients vs healthy		0.53	0.802 (0.742-0.860)	92.0	85.7	
miR-210	105 AIS patients divided into good outcome group (mRS ≤ 2, n = 40) and poor outcome group (mRS > 2, n = 65)	<72 h	Downregulated in poor outcome group	Predicting unfavorable outcome — NIHSS, mRS	0.460	0.67 (0.56-0.77)	82.5	52.3	[7]
miR-185	192 (142 AIS + 50 controls)	< 24 h	Upregulated in AIS vs controls Upregulated in poor prognosis group	Predicting unfavorable outcome — NIHSS, mRS	2.14	0.795 (0.737-0.856)	The two-miRNA combined Se and Sp reported 92.7 and 86.5		[8]
miR-424					4.08	0.841 (0.785-0.897)<br><br>Combined: 0.928 (0.870-0.993)			

miR-100-5p	103 (81 AIS + 22 controls)	<24h	Lower expression in good prognosis patients	Predicting long-term (3 month) outcome — NIHSS, mRS	5.47 ΔCT	0.887 (NR)	80	79.2	[9]
miR-140-5p	124 (62 AIS + 62 controls)	< 7 days	Higher expression in late-onset PSD group than early-onset PSD and control group	Predicting late-onset PSD — HAMD	ΔCt = −10.2	0.813 (0.715-0.910)	83	72	[10]
miR-22	257 (73 PSD group, 184 non-PSD group)	< 7 days	Upregulated in PSD group	Predicting PSD — HAMD	0.90	0.723 (0.562-0.883)	90	50	[11]
miR-221-3p	136 (76 PSD + 60 Non-PSD)	chronic	Upregulated in PSD patients	Predicting PSD — HAMD	3.21	0.900 (0.850-0.950)	78.95	91.67	[12]
miR-210	112 AIS vs 60 controls	< 3 days (n=55), 7 days (n=31) and 14 days (n=26)	Downregulated in AIS patients<br>Higher expression in good outcome group	Predicting stroke outcome — NIHSS, mRS	0.46	0.642 (0.539-0.744)	83.7	50.7	[13]

Notes: Studies with unclear miRNA collection timing do not explicitly state the timing of blood collection, but it is reasonable to presume that it was collected in the acute phase (i.e. within hours or days of hospital admission). Abbreviations: AIS, Acute ischemic stroke; BI, Barthel Index; EMVs, Endothelial Microvesicles; FSP, Favorable Stroke Patients; FSTP, favorable short-term prognosis; GCS, Glasgow Coma Scale; GOS, Glasgow Outcome Scale; HAMD, Hamilton depression scale; MMSE, Mini-Mental State Examination; MoCA, Montreal Cognitive Assessment; miR, microRNAs; mRS, Modified Rankin score; NIHSS, National Institute Health Stroke Score; NA, Not available; NFSTP, unfavorable short-term prognosis; Non-PSD, Non-Post-stroke depression; NR, not reported; PSCI, post-stroke cognitive impairment; PSCN, post-stroke cognitive normality; PSD, Post-stroke depression; rt-PA, Recombinant tissue plasminogen activator; UFSP, Unfavorable Stroke Patients.

Circulating miRNA-based biomarkers of ischemic stroke recovery

Supplemental references

1. Huang S, Zhao J, Huang D, Zhuo L, Liao S, Jiang Z. Serum miR-132 is a risk marker of post-stroke cognitive impairment. Neurosci Lett. 2016;615:102–6.

2. Lin X, Wang W, Tao T, Zhang D, Mao L, He X. Synthetic role of miR-411-5p and CT perfusion information in predicting clinical outcomes after thrombolysis in acute cerebral infarction. Acta Neurol Belg. 2022;

3. Wang Z-Q, Li K, Huang J, Huo T-T, Lv P-Y. MicroRNA Let-7i Is a Promising Serum Biomarker for Post-stroke Cognitive Impairment and Alleviated OGD-Induced Cell Damage in vitro by Regulating Bcl-2. Front Neurosci. 2020;14:215.

4. He X-W, Shi Y-H, Liu Y-S, Li G-F, Zhao R, Hu Y, et al. Increased plasma levels of miR-124-3p, miR-125b-5p and miR-192-5p are associated with outcomes in acute ischaemic stroke patients receiving thrombolysis. Atherosclerosis. 2019;289:36–43.

5. He X-W, Shi Y-H, Zhao R, Liu Y-S, Li G-F, Hu Y, et al. Plasma Levels of miR-125b-5p and miR-206 in Acute Ischemic Stroke Patients After Recanalization Treatment: A Prospective Observational Study. J Stroke Cerebrovasc Dis. 2019;28:1654–61.

6. Guo C, Zhong C, Li Q, Gao Y, Li W, Ou Y. [Expressions and neural function prognostic evaluation of serum microRNA-24 and microRNA-29b in elderly patients with acute ischemic stroke]. Zhonghua Wei Zhong Bing Ji Jiu Yi Xue. 2020;32:78–82.

7. Zeng L, Liu J, Wang Y, Wang L, Weng S, Chen S, et al. Cocktail blood biomarkers: prediction of clinical outcomes in patients with acute ischemic stroke. Eur Neurol. 2013;69:68–75.

8. Guo C, Yao Y, Li Q, Gao Y, Cao H. Expression and Clinical Value of miR-185 and miR-424 in Patients with Acute Ischemic Stroke. Int J Gen Med. 2022;15:71–8.

9. Otero-Ortega L, Alonso-López E, Pérez-Mato M, Laso-García F, Gómez-de Frutos MC, Diekhorst L, et al. Circulating Extracellular Vesicle Proteins and MicroRNA Profiles in Subcortical and Cortical-Subcortical Ischaemic Stroke. Biomedicines. 2021;9:786.

10. Liang H-B, He J-R, Tu X-Q, Ding K-Q, Yang G-Y, Zhang Y, et al. MicroRNA-140-5p: A novel circulating biomarker for early warning of late-onset post-stroke depression. J Psychiatr Res. 2019;115:129–41.

11. Hu J, Zhou W, Zhou Z, Yang Q, Xu J, Dong W. miR-22 and cerebral microbleeds in brainstem and deep area are associated with depression one month after ischemic stroke. Braz J Med Biol Res. 2020;53:e9162.

12. Cui Y, Ma G, Kong F, Song L. Diagnostic Values of miR-221-3p in Serum and Cerebrospinal Fluid for Post-Stroke Depression and Analysis of Risk Factors. Iranian Journal of Public Health [Internet]. Tehran University of Medical Sciences; 2021;50:1241.

13. Zeng L, Liu J, Wang Y, Wang L, Weng S, Tang Y, et al. MicroRNA-210 as a novel blood biomarker in acute cerebral ischemia. Front Biosci (Elite Ed). 2011;3:1265–72.
