# Supplementary material for: Circulating MicroRNAs and Extracellular Vesicle-Derived MicroRNAs as Predictors of Functional Recovery in Ischemic Stroke Patients: A Systematic Review and Meta-Analysis
Source: Int J Mol Sci. 2022 Dec 23;24(1):251. doi: 10.3390/ijms24010251 (PMC9820088; doi:10.3390/ijms24010251)
Supplement: Supplementary file 1 [file ijms-24-00251-s001.zip › Supplimental S3.pdf]

Table S3-Correlational analysis of biomarkers associated with different recovery assessment scores

Author + Year (+ref)	miRNA	miRNA source	miRNA collection time	Correlation coefficient (Spearman unless specified)	Sample size	Disability scale	Disability score timing	Observations
Ma et al, 2022 [1]	EMVs	Plasma	<24 h	0.341	72	NIHSS	At admission (within 24 h)	Pearson r Opposite correlations for total EMVs and miR-125a-5p-loaded EMVs
	miR-125a-5p-loaded EMVs			−0.276	72	NIHSS		
Peng et al., 2015 [2]	miR-let-7e	Serum	<24 h	0.29 (NS)	72	NIHSS	At admission (within 24 h)	
Wu et al., 2017 [3]	miR-23b-3p	Serum	<24 h	0.190	227	NIHSS	At admission (within 24 h)	
				0.178		mRS	At discharge (within 12 days)	
				-0.232		BI	At discharge (within 12 days)	
	miR-29b-3p	Serum	<24 h	0.181	227	NIHSS	At admission (within 24 h)	
				-0.187		BI	At discharge (within 12 days)	
	miR-181a-5p	Serum	<24 h	0.412	227	NIHSS	At admission (within 24 h)	
	miR-21–5p	Serum	<24 h	0.268	227	NIHSS	At admission (within 24 h)	
Kijpaisalratana et al. 2020 [4]	miR-125a-5p	Serum	<72 h after central vertigo occurrence due to AIS	0.239 (NS)	23	NIHSS	At admission (within 72 h)	Posterior circulation stroke Patients with central vertigo due to acute stroke
	miR-125b-5p	Serum		-0.006 (NS)		NIHSS	At admission (within 72 h)	
	miR-433-5p	Serum		0.085 (NS)		NIHSS	At admission (within 72 h)	

Circulating miRNA-based biomarkers of ischemic stroke recovery

Kautzky et al., 2022 [5]	miR-125b-5p	Plasma	On admission	0.39<br>0.56	40	NIHSS<br>mRS	Day 2<br>Day 7	Kendall τ
Zhu et al., 2019 [6]	miR-143	PBMC	< 24 h	0.203	170	NIHSS	At admission (within 24 h)	
Niu et al., 2021 [7]	miR-369-3p	Plasma	<72 h	-0.021 (NS)	453	NIHSS	At admission (within 72 h)	Exosomal
	miR-493-3p	Plasma	<72 h	-0.183	453	NIHSS	At admission (within 72 h)	Exosomal
	and miR-379-5p	Plasma	<72 h	-0.068 (NS)	453	NIHSS	At admission (within 72 h)	Exosomal
	miR-1296-5p	Plasma	<72 h	-0.185	453	NIHSS	At admission (within 72 h)	Exosomal
Li et al., 2022 [8]	miR-29b	Leukocytes	<6 h	−0.491	50	NIHSS	At admission (within 6 h)	Pearson r<br>Anterior circulation stroke
Kotb et al., 2019 [9]	miR-146a	Serum	<24 h	−0.352	44	GCS	At admission (within 24 h)	Comatose patients with severe AIS
Wu et al., 2020 [10]	miR-99b	Plasma	Unclear	−0.56	112	GOS	at day 30	-
Huang et al., 2016 [11]	miR-132	Serum	chronic	−0.859	76	MoCA	chronic	
Zhai et al., 2020 [12]	miR-195	Serum	At admission (within 72 hours)	0.655<br>−0.773	108	NIHSS<br>MoCA	At admission (within 72 hours)	-
	miR-497	Serum		0.652<br>−0.773		NIHSS<br>MoCA		
Ma et al., 2019 [13]	miR-93	Neutrophils and plasma	< 6 h	0.4124	33	BI (p=0.0211)	At admission (<6 h) + 7 days after stroke onset	miR-93 expression in plasma correlates with expression in neutrophils

Lin et al., 2022 [14]	miR-411-5p	Serum	before rt-PA, 24 h after rt-PA, and 3 months (90 days) after stroke onset	−0.470	96	NIHSS	at 24 h after thrombolysis	Correlation between miR-411-5p expression and NIHSS after thrombolysis
Xiang et al. 2017 [15]	let-7i	plasma	24 h after thrombolysis	0.28	40	NIHSS	at diagnosis	Pearson r Thrombolysis Let-7i positively correlates with NIHSS score after thrombolysis
Wang et al. 2020 [16]	let-7i	serum	Chronic (within 1 year)	0.643	36	MoCA	chronic (within 1 year)	Correlation sample included both PSCI and Non-PSCI patients
Jickling et al. 2016 [17]	let-7i	circulating leukocytes	<72h	-0.38	106	NIHSS	at diagnosis	Pearson r
Wang et al. 2019 [18]	miR-124	serum	unclear	0.374	80	NIHSS	at diagnosis (within 24 h after stroke onset)	Correlation sample included both patients and controls
Yuan et al. 2022 [19]	miR-21	serum	14 days	0.752	77	MMSE	14 days	
	miR-132	serum	14 days	0.319	77	MMSE	14 days	
	miR-200b	serum	14 days	0.379	77	MMSE	14 days	
Zhou and Qi 2021 [20]	miR-124	serum	24, 48, 72 h	0.5932	108	GOS	30 days	
Ji et al. 2016 [21]	miR-124	serum	mean 16.5 h	0.6825	65	NIHSS	at diagnosis	Exosomal
	miR-9	serum	mean 16.5 h	0.7126	65	NIHSS	at diagnosis	Exosomal
He et al.	miR-124-3p	plasma	24 h after thrombolysis	0.382	84	NIHSS	at diagnosis	Thrombolysis

2019 [22]	miR-125b-5p	plasma	24 h after thrombolysis	0.343	84	NIHSS	at diagnosis	Thrombolysis
He et al. 2019 [23]	miR-125b-5p	plasma	24 h after thrombolysis	0.262	94	NIHSS	On admission, at 24 h, on day 7	Thrombolysis
	miR-206	plasma	24 h after thrombolysis	0.341	94	NIHSS	On admission, at 24 h, on day 7	Thrombolysis miR-206 positively correlates with NIHSS scores on day 7 after thrombolysis
Qi et al. 2021 [24]	miR-124-3p	serum	2, 4, 6 h	-0.74	20	NIHSS	at diagnosis	Pearson r exosomal
Jin and Xing 2018 [25]	miR-126	plasma	<24 h	−0.402	148	NIHSS	at diagnosis	Probable patient overlap with Jin and Xing 2017 [26]
	miR-218	plasma	<24 h	0.471	148	NIHSS	at diagnosis	
	miR-130a	plasma	<24 h	−0.161 (NS)	148	NIHSS	At diagnosis	
	miR-185	plasma	<24 h	−0.129 (NS)	148	NIHSS	At diagnosis	
	miR-222	plasma	<24 h	−0.062 (NS)	148	NIHSS	At diagnosis	
Jin and Xing 2017 [26]	miR-126	plasma	<24 h	−0.398	106	NIHSS	at diagnosis	After thrombolysis
	miR-222	plasma	<24 h	0.698	106	NIHSS	at diagnosis	After thrombolysis
	miR-19a	plasma	<24 h	-0.026 (NS)	106	NIHSS	At diagnosis	After thrombolysis
	miR-296	plasma	<24 h	0.020 (NS)	106	NIHSS	At diagnosis	After thrombolysis
	miR-101	plasma	<24 h	-0.079 (NS)	106	NIHSS	At diagnosis	After thrombolysis
	miR-218	plasma	<24 h	0.099 (NS)	106	NIHSS	At diagnosis	After thrombolysis
	miR-206	plasma	<24 h	0.069 (NS)	106	NIHSS	At diagnosis	After thrombolysis
	miR-221	plasma	<24 h	0.033 (NS)	106	NIHSS	At diagnosis	After thrombolysis
	miR-378	plasma	<24 h	−0.557	106	NIHSS	at diagnosis	After thrombolysis

	miR-185	plasma	<24 h	0.171 (NS)	106	NIHSS	at diagnosis	After thrombolysis
	miR-130a	plasma	<24 h	0.013 (NS)	106	NIHSS	at diagnosis	After thrombolysis
Chen et al. 2020 [27]	miR-195	plasma	<24 h	−0.139	215	NIHSS	at diagnosis	-
	miR-9	plasma	<24 h	−0.335	215	NIHSS	at diagnosis	
Yang et al. 2018 [28]	miR-195	plasma	<72 h	−0.3684	96	NIHSS	at diagnosis	
Liu et al. 2021 [29]	miR-21	PBMCs+serum	at diagnosis	−0.284	170	NIHSS	at diagnosis	
Zhou and Zhang 2014 [30]	miR-21	plasma	at diagnosis	−0.703	68	NIHSS	at diagnosis	
Wang et al. 2015 [31]	miR-29b	WBCs	<72 h	−0.349	58	NIHSS	at 3 months	
Ma et al. 2022 [32]	miR-29b	neutrophils	<6 h	−0.547	60	NIHSS	at diagnosis	
Abdelaleem et al. 2022 [33]	miR-9	serum	unclear	0.681	77	NIHSS	at diagnosis	Patients with stroke and type 2 diabetes
Xue et al. 2018 [34]	miR-9	serum	unclear	0.627	65	NIHSS	at diagnosis	
Xie et al. 2019 [35]	miR-124	plasma	unclear	−0.567	40	NIHSS	unclear	
	miR-24	Serum	unclear	−0.758	170	NIHSS	unclear	

Guo et al., 2020 [36]	miR-29b	Serum	NA (abstract only)	−0.794	170	NIHSS	NA (abstract only)	Pearson r
Yuan et al., 2016 [37]	miR-335	Plasma	< 24 h following patient admission	−0.680	152	NIHSS	at admission	Pearson r<br>Time from onset to admission <14 days
Yuan et al., 2016 [38]	miR-26b	Plasma	< 24 h following patient admission	−0.660	152	NIHSS	at admission	Pearson r<br>Time from onset to admission <14 days
Zhao et al., 2016 [39]	miR-335	Plasma	< 24 h	−0.3682	168	NIHSS	At admission	Pearson r
Zhong et al., 2021 [40]	miR-497	Serum	At admission and at discharge	−0.611	128	NIHSS	At admission and clinical discharge	Intravenous thrombolysis or mechanical thrombectomy
Song et al., 2021 [41]	miR-409-3p	Serum	<9 h	0.865	80	NIHSS	Unclear	
Yang et al., 2020 [42]	miR-135b	Serum	< 24 h and at day 14 after stroke onset	−0.835	79	NIHSS	Unclear	
Liu et al., 2019 [43]	miR-128	Lymphocytes	< 72 h	0.3944<br>0.4357	40	NIHSS<br>mRS	at 7 days<br>at 90 days	
Zhao et al., 2020 [44]	miR-494	Lymphocytes	< 6h	0.3339	43	NIHSS	<6h	
Sheikhbahaei et al., 2019 [45]	MiR-503	Serum	< 72 h + 3 months	0.45<br>0.48	50	NIHSS<br>acute phase mRS	< 72 h<br>< 72 h	Correlation sample included patients with diabetes and without stroke, diabetic patients with stroke, stroke patients without diabetes<br>Higher disability at 3 months for patients

								with higher initial expression of miR-503
Guo et al., 2022 [46]	miR-185	Serum	<24 h	0.735<br><br>0.802	142	NIHSS<br><br>mRS	<24h<br><br><24h	
	miR-424	Serum	<24 h	0.796<br><br>0.873	142	NIHSS<br><br>mRS	<24h<br><br><24h	
Fu et al., 2019 [47]	miR-451	Unknown	<12 hours	0.6104	108	NIHSS	3 months	
Ye et al., 2021 [48]	miRNA-27-3p	Serum	On hospital admission	0.7320	86	NIHSS	On hospital admission	Exosomal
Yang et al, 2016 [49]	miR-107	Plasma	<24 h	0.52	114	NIHSS	<24 h	
	miR-128b	Plasma	<24 h	0.44	114	NIHSS	<24 h	
	miR-153	Plasma	<24 h	0.35	114	NIHSS	<24 h	
Zhou et al., 2018 [50]	miR-134	Serum	at 24-, 48- and 72 h	0.6079	50	NIHSS	At admission (<24 h)	Exosomal
Chen et al., 2018 [51]	miR-146b	Serum	< 24 h	0.6460	128	NIHSS	at admission (<24 h) and 24 hours after stroke onset	
Chen et al., 2017 [52]	miR-223	Serum	< 72 h	0.31	50	NIHSS	at admission (<72 h)	Exosomal
Liang et al., 2016 **[53]**	mir-34a-5p	Plasma	< 12 h	−0.692	102	NIHSS	At admission	
Wang et al., 2014 **[54]**	miR-223	Leucocytes	At 24, 48, 72 h	−0.531	79	NIHSS	At admission (<72 h)	
	miR-9-5p	Serum	<6 h	0.066	88	mRS	At admission (<6 h)	

Wang et al., 2021 **[55]**	miR-128-3p	Serum	<6 h	0.082	88	mRS	At admission (<6 h)
Jia et al., 2015 **[56]**	miR-145	Serum	<24 h	0.6288	146	NIHSS	At admission and 24 h after stroke onset
Liang et al., 2019 [57]	miR-140-5p	Plasma	<24 h	0.4625	62	HAMD	3 months
Hu et. al, 2020 [58]	miR-22	Plasma	< 7 days	0.635	73	HAMD	1 month
Cui et al., 2021 [59]	miR-221-3p	Serum	Chronic (unknown)	0.620	136	NIHSS	Chronic (unknown)
				0.608	136	HAMD	Chronic (unknown)

Notes: Jin and Xing 2018 is a follow-up study of Jin and Xing 2017, overlapping study periods, being performed in the same medical center and having very similar results (I^2=0%). We presume that patients from the first study were included in the second. Studies with *unclear* miRNA collection timing do not explicitly state the timing of blood collection, but it is reasonable to presume that it was collected in the acute phase (i.e. within hours or days of hospital admission). Abbreviations: AIS, Acute ischemic stroke; BI, Barthel Index; EMVs, Endothelial Microvesicles; GCS, Glasgow Coma Scale; GOS, Glasgow Outcome Scale; MMSE, Mini-Mental State Examination; MoCA, Montreal Cognitive Assessment; miR, microRNAs; mRS, Modified Rankin score; NIHSS, National Institute Health Stroke Score

Supplemental references

1. Ma X, Liao X, Liu J, Wang Y, Wang X, Chen Y, et al. Circulating endothelial microvesicles and their carried miR-125a-5p: potential biomarkers for ischaemic stroke. Stroke Vasc Neurol.; 2022;svn-2021-001476

2. Peng G, Yuan Y, Wu S, He F, Hu Y, Luo B. MicroRNA let-7e Is a Potential Circulating Biomarker of Acute Stage Ischemic Stroke. Transl Stroke Res. 2015;6:437–45.

3. Wu J, Fan C-L, Ma L-J, Liu T, Wang C, Song J-X, et al. Distinctive expression signatures of serum microRNAs in ischaemic stroke and transient ischaemic attack patients. Thromb Haemost. 2017;117:992–1001.

4. Kijpaisalratana N, Nimsamer P, Khamwut A, Payungporn S, Pisitkun T, Chutinet A, et al. Serum miRNA125a-5p, miR-125b-5p, and miR-433-5p as biomarkers to differentiate between posterior circulation stroke and peripheral vertigo. BMC Neurology. 2020;20:372.

5. Kautzky V. Identifizierung von Einflussfaktoren auf die Konzentrationen der zirkulierenden microRNAs miR-125a-5p, miR-125b-5p und miR-143-3p nach ischämischem Schlaganfall [PhDThesis]. Ludwig-Maximilians-Universität München; 2022.

6. Zhu X, Ding J, Wang B, Wang J, Xu M. Circular RNA DLGAP4 is down-regulated and negatively correlates with severity, inflammatory cytokine expression and pro-inflammatory gene miR-143 expression in acute ischemic stroke patients. Int J Clin Exp Pathol. 2019;12:941–8.

7. Niu M, Li H, Li X, Yan X, Ma A, Pan X, et al. Circulating Exosomal miRNAs as Novel Biomarkers Perform Superior Diagnostic Efficiency Compared With Plasma miRNAs for Large-Artery Atherosclerosis Stroke. Front Pharmacol. 2021;12:791644.

8. Li G, Ma X, Zhao H, Fan J, Liu T, Luo Y, et al. Long non-coding RNA H19 promotes leukocyte inflammation in ischemic stroke by targeting the miR-29b/C1QTNF6 axis. CNS Neuroscience & Therapeutics. 2022;28:953–63.

9. Kotb HG, Ibrahim AH, Mohamed EF, Ali OM, Hassanein N, Badawy D, et al. The expression of microRNA 146a in patients with ischemic stroke: an observational study. Int J Gen Med. 2019;12:273–8.

10. Wu X, Zhang X, Li D, Zhu Z. Plasma level of miR-99b may serve as potential diagnostic and short-term prognostic markers in patients with acute cerebral infarction. J Clin Lab Anal. 2020;34:e23093.

11. Huang S, Zhao J, Huang D, Zhuo L, Liao S, Jiang Z. Serum miR-132 is a risk marker of post-stroke cognitive impairment. Neurosci Lett. 2016;615:102–6.

12. Zhai Y, Zhu Z, Li H, Zhao C, Huang Y, Wang P. miR-195 and miR-497 in acute stroke and their correlations with post-stroke cognitive impairment. Int J Clin Exp Pathol. 2020;13:3092–9.

13. Ma Q, Li G, Tao Z, Wang J, Wang R, Liu P, et al. Blood microRNA-93 as an indicator for diagnosis and prediction of functional recovery of acute stroke patients. J Clin Neurosci. 2019;62:121–7.

14. Lin X, Wang W, Tao T, Zhang D, Mao L, He X. Synthetic role of miR-411-5p and CT perfusion information in predicting clinical outcomes after thrombolysis in acute cerebral infarction. Acta Neurol Belg. 2022;

15. Xiang W, Tian C, Lin J, Wu X, Pang G, Zhou L, et al. Plasma let-7i and miR-15a expression are associated with the effect of recombinant tissue plasminogen activator treatment in acute ischemic stroke patients. Thromb Res. 2017;158:121–5.

16. Wang Z-Q, Li K, Huang J, Huo T-T, Lv P-Y. MicroRNA Let-7i Is a Promising Serum Biomarker for Post-stroke Cognitive Impairment and Alleviated OGD-Induced Cell Damage in vitro by Regulating Bcl-2. Front Neurosci. 2020;14:215.

17. Jickling GC, Ander BP, Shroff N, Orantia M, Stamova B, Dykstra-Aiello C, et al. Leukocyte response is regulated by microRNA let7i in patients with acute ischemic stroke. Neurology. 2016;87:2198–205.

18. Wang J, Huang Q, Ding J, Wang X. Elevated serum levels of brain-derived neurotrophic factor and miR-124 in acute ischemic stroke patients and the molecular mechanism. 3 Biotech. 2019;9:386.

19. Yuan M, Guo Y-S, Zhang X-X, Gao Z-K, Shen X-Y, Han Y, et al. Diagnostic performance of miR-21, miR-124, miR-132, and miR-200b serums in post-stroke cognitive impairment patients. Folia Neuropathol. 2022;60:228–36.

20. Zhou X, Qi L. miR-124 Is Downregulated in Serum of Acute Cerebral Infarct Patients and Shows Diagnostic and Prognostic Value. Clin Appl Thromb Hemost. 2021;27:10760296211035446.

21. Ji Q, Ji Y, Peng J, Zhou X, Chen X, Zhao H, et al. Increased Brain-Specific MiR-9 and MiR-124 in the Serum Exosomes of Acute Ischemic Stroke Patients. PLoS One. 2016;11:e0163645.

22. He X-W, Shi Y-H, Liu Y-S, Li G-F, Zhao R, Hu Y, et al. Increased plasma levels of miR-124-3p, miR-125b-5p and miR-192-5p are associated with outcomes in acute ischaemic stroke patients receiving thrombolysis. Atherosclerosis. 2019;289:36–43.

23. He X-W, Shi Y-H, Zhao R, Liu Y-S, Li G-F, Hu Y, et al. Plasma Levels of miR-125b-5p and miR-206 in Acute Ischemic Stroke Patients After Recanalization Treatment: A Prospective Observational Study. J Stroke Cerebrovasc Dis. 2019;28:1654–61.

24. Qi Z, Zhao Y, Su Y, Cao B, Yang J-J, Xing Q. Serum Extracellular Vesicle–Derived miR-124-3p as a Diagnostic and Predictive Marker for Early-Stage Acute Ischemic Stroke. Frontiers in Molecular Biosciences. 2021;8:685088

25. Jin F, Xing J. Circulating miR-126 and miR-130a levels correlate with lower disease risk, disease severity, and reduced inflammatory cytokine levels in acute ischemic stroke patients. Neurol Sci. 2018;39:1757–65.

26. Jin F, Xing J. Circulating pro-angiogenic and anti-angiogenic microRNA expressions in patients with acute ischemic stroke and their association with disease severity. Neurol Sci. 2017;38:2015–23.

27. Chen X, Zhang X, Su C, Huang S. Long noncoding RNA HULC in acute ischemic stroke: Association with disease risk, severity, and recurrence-free survival and relation with IL-6, ICAM1, miR-9, and miR-195. J Clin Lab Anal. 2020;34:e23500.

28. Yang G, Liu Z, Wang L, Chen X, Wang X, Dong Q, et al. MicroRNA-195 protection against focal cerebral ischemia by targeting CX3CR1. J Neurosurg. 2018;1–10.

29. Liu C, Huang H, Li Y, Zhao H. The relationship of long non-coding RNA maternally expressed gene 3 with microRNA-21 and their correlation with acute ischemic stroke risk, disease severity and recurrence risk. Clin Neurol Neurosurg. 2021;210:106940.

30. Zhou J, Zhang J. Identification of miRNA-21 and miRNA-24 in plasma as potential early stage markers of acute cerebral infarction. Mol Med Rep. 2014;10:971–6.

31. Wang Y, Huang J, Ma Y, Tang G, Liu Y, Chen X, et al. MicroRNA-29b is a therapeutic target in cerebral ischemia associated with aquaporin 4. J Cereb Blood Flow Metab. 2015;35:1977–84.

32. Ma X, Yun HJ, Elkin K, Guo Y, Ding Y, Li G. MicroRNA-29b Suppresses Inflammation and Protects Blood-Brain Barrier Integrity in Ischemic Stroke. Mediators of Inflammation. Hindawi; 2022;2022:e1755416.

33. Abdelaleem OO, Shaker OG, Mohamed MM, Ahmed TI, Elkhateeb AF, Abdelghaffar NK, et al. Differential Expression of Serum TUG1, LINC00657, miR-9, and miR-106a in Diabetic Patients With and Without Ischemic Stroke. Frontiers in Molecular Biosciences. 2022;8.

34. Xue Y, Li M, Liu D, Zhu Q, Chen H. Expression of miR-9 in the serum of patients with acute ischemic stroke and its effect on neuronal damage. Int J Clin Exp Pathol. Madison: E-Century Publishing Corp; 2018;11:5885–92.

35. Xie Z, Liu B, Zhou M, Chen Y. Expression and clinical significance of plasma miR-124 in acute ischemic stroke. The Journal of Practical Medicine. 2019;343–5.

36. Guo C, Zhong C, Li Q, Gao Y, Li W, Ou Y. [Expressions and neural function prognostic evaluation of serum microRNA-24 and microRNA-29b in elderly patients with acute ischemic stroke]. Zhonghua Wei Zhong Bing Ji Jiu Yi Xue. 2020;32:78–82.

37. Yuan M, Yuan H, Zhou C, Liu F, Lin C, Tang Y. The significance of low plasma miR-335 level in patients with acute cerebral infarction may be associated with the loss of control of CALM1 expression. Int J Clin Exp Med. Madison: E-Century Publishing Corp; 2016;9:19595–601.

38. Yuan M, Tang Y, Zhou C, Liu F, Chen L, Yuan H. Elevated plasma CaM expression in patients with acute cerebral infarction predicts poor outcomes and is inversely associated with miR-26b expression. Int J Neurosci. 2016;126:408–14.

39. Zhao B, Zhu Z, Hao J, Wan Z, Guo X. Decreased plasma miR-335 expression in patients with acute ischemic stroke and its association with calmodulin expression. J Int Med Res. 2016;44:1331–8.

40. Zhong C, Yin C, Niu G, Ning L, Pan J. MicroRNA miR-497 is closely associated with poor prognosis in patients with cerebral ischemic stroke. Bioengineered. 2021;12:2851–62.

41. Song X-D, Li S-X, Zhu M. Plasma miR-409-3p promotes acute cerebral infarction via suppressing CTRP3. Kaohsiung J Med Sci. 2021;37:324–33.

42. Yang S, Zhan X, He M, Wang J, Qiu X. miR-135b levels in the peripheral blood serve as a marker associated with acute ischemic stroke. Exp Ther Med. 2020;19:3551–8.

43. Liu P, Han Z, Ma Q, Liu T, Wang R, Tao Z, et al. Upregulation of MicroRNA-128 in the Peripheral Blood of Acute Ischemic Stroke Patients is Correlated with Stroke Severity Partially through Inhibition of Neuronal Cell Cycle Reentry. Cell Transplant. 2019;28:839–50.

44. Zhao H, Li G, Wang R, Tao Z, Ma Q, Zhang S, et al. Silencing of microRNA-494 inhibits the neurotoxic Th1 shift via regulating HDAC2-STAT4 cascade in ischaemic stroke. Br J Pharmacol. 2020;177:128–44.

45. Sheikhbahaei S, Manizheh D, Mohammad S, Hasan TM, Saman N, Laleh R, et al. Can MiR-503 be used as a marker in diabetic patients with ischemic stroke? BMC Endocr Disord. 2019;19:42.

46. Guo C, Yao Y, Li Q, Gao Y, Cao H. Expression and Clinical Value of miR-185 and miR-424 in Patients with Acute Ischemic Stroke. Int J Gen Med. 2022;15:71–8.

47. Fu C, Chen S, Cai N, Liu Z, Wang P, Zhao J. Potential Neuroprotective Effect of miR-451 Against Cerebral Ischemia/Reperfusion Injury in Stroke Patients and a Mouse Model. World Neurosurg. 2019;130:e54–61.

48. Ye Z, Hu J, Xu H, Sun B, Jin Y, Zhang Y, et al. Serum Exosomal microRNA-27-3p Aggravates Cerebral Injury and Inflammation in Patients with Acute Cerebral Infarction by Targeting PPARγ. Inflammation. 2021;44:1035–48.

49. Yang Z-B, Li T-B, Zhang Z, Ren K-D, Zheng Z-F, Peng J, et al. The Diagnostic Value of Circulating Brain-specific MicroRNAs for Ischemic Stroke. Intern Med. 2016;55:1279–86.

50. Zhou J, Chen L, Chen B, Huang S, Zeng C, Wu H, et al. Increased serum exosomal miR-134 expression in the acute ischemic stroke patients. BMC Neurol. 2018;18:198.

51. Chen Z, Wang K, Huang J, Zheng G, Lv Y, Luo N, et al. Upregulated Serum MiR-146b Serves as a Biomarker for Acute Ischemic Stroke. Cell Physiol Biochem. 2018;45:397–405.

52. Chen Y, Song Y, Huang J, Qu M, Zhang Y, Geng J, et al. Increased Circulating Exosomal miRNA-223 Is Associated with Acute Ischemic Stroke. Front Neurol. 2017;8:57.

53. Liang T, Lou J. Increased Expression of mir-34a-5p and Clinical Association in Acute Ischemic Stroke Patients and in a Rat Model. Med Sci Monit. 2016;22:2950–5.

54. Wang Y, Zhang Y, Huang J, Chen X, Gu X, Wang Y, et al. Increase of circulating miR-223 and insulin-like growth factor-1 is associated with the pathogenesis of acute ischemic stroke in patients. BMC Neurol. 2014;14:77.

55. Wang Q, Wang F, Fu F, Liu J, Sun W, Chen Y. Diagnostic and prognostic value of serum miR-9-5p and miR-128-3p levels in early-stage acute ischemic stroke. Clinics (Sao Paulo). 2021;76:e2958.

56. Jia L, Hao F, Wang W, Qu Y. Circulating miR-145 is associated with plasma high-sensitivity C-reactive protein in acute ischemic stroke patients. Cell Biochemistry and Function. 2015;33:314–9.

57. Liang H-B, He J-R, Tu X-Q, Ding K-Q, Yang G-Y, Zhang Y, et al. MicroRNA-140-5p: A novel circulating biomarker for early warning of late-onset post-stroke depression. J Psychiatr Res. 2019;115:129–41.

58. Hu J, Zhou W, Zhou Z, Yang Q, Xu J, Dong W. miR-22 and cerebral microbleeds in brainstem and deep area are associated with depression one month after ischemic stroke. Braz J Med Biol Res. 2020;53:e9162.

59. Cui Y, Ma G, Kong F, Song L. Diagnostic Values of miR-221-3p in Serum and Cerebrospinal Fluid for Post-Stroke Depression and Analysis of Risk Factors. Iranian Journal of Public Health. Tehran University of Medical Sciences; 2021;50:1241.
