# Supplementary material for: Circulating MicroRNAs and Extracellular Vesicle-Derived MicroRNAs as Predictors of Functional Recovery in Ischemic Stroke Patients: A Systematic Review and Meta-Analysis
Source: Int J Mol Sci. 2022 Dec 23;24(1):251. doi: 10.3390/ijms24010251 (PMC9820088; doi:10.3390/ijms24010251)
Supplement: Supplementary file 1 [file ijms-24-00251-s001.zip › Supplimental S4.pdf]

Circulating miRNA-based biomarkers of ischemic stroke recovery
Table S4

Let-7i	Xiang et al. 2017 (1)	Wang et al. 2020 (2)	Jickling et al. 2016 (3)
Prospective	Yes	Yes	Yes
Biomarker evaluator blinded to outcome	No	No	No
Defined time period	Yes	No	Yes
Pre-defined clinical endpoints	Yes	Yes	Yes
Sample size calculation	No	No	No
Candidate variables	No	No	Yes
Measurement method described	Yes	Yes	Yes
Inclusion/exclusion criteria	Yes	Yes	Yes
**Score (of 8)**	5	4	6

miR-124	Wang et al. 2019 (4)	Yuan et al. 2022 (5)	Xie et al. 2019 (6)	Zhou and Qi 2021 (7)	Ji et al. 2016 (8)
Prospective	Yes	Yes	NA (abstract only)	Yes	Yes
Biomarker evaluator blinded to outcome	No	No		No	No
Defined time period	No	Yes		Yes	Yes
Pre-defined clinical endpoints	Yes	Yes		Yes	Yes
Sample size calculation	No	No		No	No
Candidate variables	No	No		Yes	No
Measurement method described	Yes	Yes		Yes	Yes
Inclusion/exclusion criteria	Yes	Yes		Yes	No
**Score (of 8)**	4	5		6	4

miR-124-3p	He et al. 2019 (9)	Qi et al. 2021 (10)
Prospective	Yes	No
Biomarker evaluator blinded to outcome	No	No
Defined time period	Yes	Yes
Pre-defined clinical endpoints	Yes	Yes
Sample size calculation	No	No
Candidate variables	No	No
Measurement method described	Yes	Yes
Inclusion/exclusion criteria	Yes	No
**Score (of 8)**	5	3

miR-126	Jin and Xing 2017 (11)	Jin and Xing 2018 (12)

Prospective	Yes	Yes
Biomarker evaluator blinded to outcome	No	No
Defined time period	Yes	Yes
Pre-defined clinical endpoints	Yes	Yes
Sample size calculation	No	No
Candidate variables	Yes	Yes
Measurement method described	Yes	Yes
Inclusion/exclusion criteria	Yes	Yes
**Score (of 8)**	6	6

miR-195	Chen et al. 2020 (13)	Yang et al. 2018 (14)	Zhai et al. 2020 (15)
Prospective	Yes	Yes	Yes
Biomarker evaluator blinded to outcome	No	No	No
Defined time period	Yes	Yes	Yes
Pre-defined clinical endpoints	Yes	Yes	Yes
Sample size calculation	No	No	No
Candidate variables	No	No	Yes
Measurement method described	Yes	Yes	Yes
Inclusion/exclusion criteria	Yes	No	Yes
**Score (of 8)**	5	4	6

miR-21	Liu et al. 2021 (16)	Zhou and Zhang 2014 (17)	Yuan et al. 2022 (5)
Prospective	Yes	Yes	Yes
Biomarker evaluator blinded to outcome	No	No	No
Defined time period	Yes	Yes	Yes
Pre-defined clinical endpoints	Yes	Yes	Yes
Sample size calculation	No	No	No
Candidate variables	No	No	No
Measurement method described	Yes	Yes	Yes
Inclusion/exclusion criteria	Yes	Yes	Yes
**Score (of 8)**	5	5	5

miR-29b	Wang et al. 2015 (18)	Guo et al. 2020 (19)	Ma et al. 2022 (20)	Li et al. 2022 (21)
Prospective	Yes	NA (Abstract only)	No	Yes
Biomarker evaluator blinded to outcome	No		Yes	No
Defined time period	Yes		Yes	No
Pre-defined clinical endpoints	Yes		Yes	Yes
Sample size calculation	No		No	No
Candidate variables	Yes		No	No

Measurement method described	Yes		Yes	Yes
Inclusion/exclusion criteria	Yes		No	No
**Score (of 8)**	6		4	37.5

miR-9	Chen et al. 2020 (13)	Abdelaleem et al. 2012 (22)	Xue al. 2018 (23)	Ji et al. 2016 (8)
Prospective	Yes	Yes	Yes	Yes
Biomarker evaluator blinded to outcome	No	No	Yes	No
Defined time period	Yes	No	No	Yes
Pre-defined clinical endpoints	Yes	Yes	Yes	Yes
Sample size calculation	No	No	No	No
Candidate variables	No	Yes	No	No
Measurement method described	Yes	Yes	Yes	Yes
Inclusion/exclusion criteria	Yes	Yes	No	No
**Score (of 8)**	5	5	4	4

miR-125b-5p	Kautzky et al. 2022 (24)	He et al. 2019 (25)
Prospective	Yes	Yes
Biomarker evaluator blinded to outcome	No	No
Defined time period	Yes	Yes
Pre-defined clinical endpoints	Yes	Yes
Sample size calculation	No	No
Candidate variables	Yes	No
Measurement method described	Yes	Yes
Inclusion/exclusion criteria	Yes	Yes
**Score (of 8)**	6	62.5

miR-335	Yuan et al. 2016 (26)	Zhao et al. 2016 (27)
Prospective	Yes	Yes
Biomarker evaluator blinded to outcome	No	No
Defined time period	Yes	Yes
Pre-defined clinical endpoints	Yes	Yes
Sample size calculation	No	No
Candidate variables	No	Yes
Measurement method described	Yes	Yes
Inclusion/exclusion criteria	Yes	Yes
**Score (of 8)**	5	6

miR-185	Guo et al. 2022 (28)
Prospective	Yes
Biomarker evaluator blinded to outcome	No
Defined time period	Yes
Pre-defined clinical endpoints	Yes
Sample size calculation	No
Candidate variables	Yes
Measurement method described	Yes
Inclusion/exclusion criteria	Yes
**Score (of 8)**	75

miR-125a-5p	Kijpaisalratana et al. 2020 (29)	Ma et al. 2022 (30)
Prospective	Yes	Yes
Biomarker evaluator blinded to outcome	No	No
Defined time period	Yes	Yes
Pre-defined clinical endpoints	Yes	Yes
Sample size calculation	No	Yes
Candidate variables	Yes	Yes
Measurement method described	Yes	Yes
Inclusion/exclusion criteria	Yes	Ye
**Score (of 8)**	75	87.5

Let-7e	Peng et al. 2015 (31)
Prospective	Yes
Biomarker evaluator blinded to outcome	No
Defined time period	Yes
Pre-defined clinical endpoints	Yes
Sample size calculation	No
Candidate variables	Yes
Measurement method described	Yes

Inclusion/exclusion criteria	Yes
**Score (of 8)**	75

Multiple exosomal-derived miRNAs	Wu et al. 2017 (32)	Zeng et al. 2013 (33)	Otero-Ortega et al. 2021 (34)	Yang et al. 2016 (35)
Prospective	Yes	Yes	Yes	Yes
Biomarker evaluator blinded to outcome	No	No	No	No
Defined time period	Yes	Yes	Yes	Yes
Pre-defined clinical endpoints	Yes	Yes	Yes	Yes
Sample size calculation	No	No	No	No
Candidate variables	No	Yes	No	No
Measurement method described	Yes	Yes	Yes	Yes
Inclusion/exclusion criteria	Yes	Yes	Yes	Yes
**Score (of 8)**	62.5	75	62.5	62.5

Mir-143	Zhu et al. 2019 (36)
Prospective	Yes
Biomarker evaluator blinded to outcome	No
Defined time period	Yes
Pre-defined clinical endpoints	Yes
Sample size calculation	No
Candidate variables	Yes
Measurement method described	Yes
Inclusion/exclusion criteria	Yes
**Score (of 8)**	75

Circulating Exosomal miRNA	Niu et al. 2021 (37)
Prospective	Yes
Biomarker evaluator blinded to outcome	No
Defined time period	Yes
Pre-defined clinical endpoints	Yes
Sample size calculation	No
Candidate variables	No

Measurement method described	Yes
Inclusion/exclusion criteria	Yes
**Score (of 8)**	62.5

Mir-132	Huang et al. 2016 (38)
Prospective	Yes
Biomarker evaluator blinded to outcome	No
Defined time period	Yes
Pre-defined clinical endpoints	Yes
Sample size calculation	No
Candidate variables	No
Measurement method described	Yes
Inclusion/exclusion criteria	Yes
**Score (of 8)**	62.5

Mir-411-5p	Lin et al. 2022 (39)
Prospective	Yes
Biomarker evaluator blinded to outcome	No
Defined time period	Yes
Pre-defined clinical endpoints	Yes
Sample size calculation	No
Candidate variables	No
Measurement method described	Yes
Inclusion/exclusion criteria	Yes
**Score (of 8)**	62.5

Mir-26b	Yuan et al. 2016 (40)
Prospective	Yes
Biomarker evaluator blinded to outcome	No
Defined time period	No
Pre-defined clinical endpoints	Yes
Sample size calculation	No
Candidate variables	No

Measurement method described	Yes
Inclusion/exclusion criteria	Yes
**Score (of 8)**	50

Mir-497	Zhong et al. 2021 (41)	Zhai et al. 2020 (15)
Prospective	Yes	Yes
Biomarker evaluator blinded to outcome	No	No
Defined time period	No	Yes
Pre-defined clinical endpoints	Yes	Yes
Sample size calculation	No	No
Candidate variables	No	Yes
Measurement method described	Yes	Yes
Inclusion/exclusion criteria	Yes	Yes
**Score (of 8)**	50	6

Mir-409-3p	Song et al. 2021 (42)
Prospective	Yes
Biomarker evaluator blinded to outcome	No
Defined time period	Yes
Pre-defined clinical endpoints	Yes
Sample size calculation	No
Candidate variables	No
Measurement method described	Yes
Inclusion/exclusion criteria	Yes
**Score (of 8)**	62.5

Mir-155	Zhang et al. 2020 (43)
Prospective	Yes
Biomarker evaluator blinded to outcome	No
Defined time period	Yes
Pre-defined clinical endpoints	Yes
Sample size calculation	Yes

Candidate variables	Yes
Measurement method described	Yes
Inclusion/exclusion criteria	Yes
**Score (of 8)**	87.5

Mir-135b	Yang et al. 2020 (44)
Prospective	Yes
Biomarker evaluator blinded to outcome	No
Defined time period	Yes
Pre-defined clinical endpoints	Yes
Sample size calculation	No
Candidate variables	Yes
Measurement method described	Yes
Inclusion/exclusion criteria	Yes
**Score (of 8)**	75

Mir-128	Liu et al. 2019 (45)
Prospective	Yes
Biomarker evaluator blinded to outcome	No
Defined time period	Yes
Pre-defined clinical endpoints	Yes
Sample size calculation	No
Candidate variables	Yes
Measurement method described	Yes
Inclusion/exclusion criteria	Yes
**Score (of 8)**	75

Mir-494	Zhao et al. 2020 (46)
Prospective	Yes
Biomarker evaluator blinded to outcome	No
Defined time period	No
Pre-defined clinical endpoints	Yes
Sample size calculation	No

Candidate variables	No
Measurement method described	Yes
Inclusion/exclusion criteria	Yes
**Score (of 8)**	50

Mir-503	Sheikhbahaei et al. 2019 (47)
Prospective	Yes
Biomarker evaluator blinded to outcome	No
Defined time period	No
Pre-defined clinical endpoints	Yes
Sample size calculation	No
Candidate variables	Yes
Measurement method described	Yes
Inclusion/exclusion criteria	Yes
**Score (of 8)**	62.5

Mir-451	Fu et al. 2019 (48)
Prospective	Yes
Biomarker evaluator blinded to outcome	No
Defined time period	No
Pre-defined clinical endpoints	Yes
Sample size calculation	No
Candidate variables	Yes
Measurement method described	Yes
Inclusion/exclusion criteria	Yes
**Score (of 8)**	62.5

Mir-27-3p	Ye et al. 2021 (49)
Prospective	Yes
Biomarker evaluator blinded to outcome	No
Defined time period	No
Pre-defined clinical endpoints	Yes

Sample size calculation	No
Candidate variables	No
Measurement method described	Yes
Inclusion/exclusion criteria	No
**Score (of 8)**	37.5

Mir-134	Zhou et al. 2018 (50)
Prospective	Yes
Biomarker evaluator blinded to outcome	No
Defined time period	Yes
Pre-defined clinical endpoints	Yes
Sample size calculation	No
Candidate variables	No
Measurement method described	Yes
Inclusion/exclusion criteria	Yes
**Score (of 8)**	62.5

Mir-146b	Chen et al. 2018 (51)
Prospective	Yes
Biomarker evaluator blinded to outcome	No
Defined time period	Yes
Pre-defined clinical endpoints	Yes
Sample size calculation	No
Candidate variables	No
Measurement method described	Yes
Inclusion/exclusion criteria	Yes
**Score (of 8)**	62.5

Mir-223	Chen et al. 2017 (52)	Wang et al. 2014 (53)
Prospective	Yes	Yes
Biomarker evaluator blinded to outcome	No	No
Defined time period	No	Yes
Pre-defined clinical endpoints	Yes	Yes

Sample size calculation	No	No
Candidate variables	Yes	Yes
Measurement method described	Yes	Yes
Inclusion/exclusion criteria	Yes	Yes
**Score (of 8)**	62.5	75

Mir-34a-5p	Liang et al. 2016 (54)
Prospective	Yes
Biomarker evaluator blinded to outcome	No
Defined time period	No
Pre-defined clinical endpoints	Yes
Sample size calculation	No
Candidate variables	No
Measurement method described	Yes
Inclusion/exclusion criteria	No
**Score (of 8)**	37.5

Mir-9-5p	Wang et al. 2021 (55)
Prospective	Yes
Biomarker evaluator blinded to outcome	No
Defined time period	No
Pre-defined clinical endpoints	Yes
Sample size calculation	No
Candidate variables	No
Measurement method described	Yes
Inclusion/exclusion criteria	Yes
**Score (of 8)**	50

Mir-145	Jia et al. 2015 (56)
Prospective	Yes
Biomarker evaluator blinded to outcome	No
Defined time period	Yes
Pre-defined clinical endpoints	Yes

Sample size calculation	No
Candidate variables	No
Measurement method described	Yes
Inclusion/exclusion criteria	Yes
**Score (of 8)**	62.5

Mir-140-5p	Liang et al. 2019 (57)
Prospective	Yes
Biomarker evaluator blinded to outcome	No
Defined time period	Yes
Pre-defined clinical endpoints	Yes
Sample size calculation	No
Candidate variables	Yes
Measurement method described	Yes
Inclusion/exclusion criteria	Yes
**Score (of 8)**	75

Mir-22	Hu et al. 2020 (58)
Prospective	Yes
Biomarker evaluator blinded to outcome	No
Defined time period	No
Pre-defined clinical endpoints	Yes
Sample size calculation	No
Candidate variables	Yes
Measurement method described	Yes
Inclusion/exclusion criteria	Yes
**Score (of 8)**	62.5

Mir-221-3p	Cui et al. 2021 (59)
Prospective	No
Biomarker evaluator blinded to outcome	No
Defined time period	No
Pre-defined clinical endpoints	Yes

Sample size calculation	No
Candidate variables	Yes
Measurement method described	No
Inclusion/exclusion criteria	Yes
**Score (of 8)**	37.5

Mir-210	Zeng et al. 2011 (60)
Prospective	Yes
Biomarker evaluator blinded to outcome	No
Defined time period	No
Pre-defined clinical endpoints	Yes
Sample size calculation	No
Candidate variables	No
Measurement method described	Yes
Inclusion/exclusion criteria	Yes
**Score (of 8)**	50

Mir-146a	Kotb et al., 2019 (61)
Prospective	Yes
Biomarker evaluator blinded to outcome	No
Defined time period	yes
Pre-defined clinical endpoints	Yes
Sample size calculation	No
Candidate variables	No
Measurement method described	Yes
Inclusion/exclusion criteria	Yes
**Score (of 8)**	62.5

Mir-99b	Wu et al., 2020 (62)
Prospective	Yes
Biomarker evaluator blinded to outcome	No
Defined time period	yes
Pre-defined clinical endpoints	Yes

Sample size calculation	No
Candidate variables	No
Measurement method described	Yes
Inclusion/exclusion criteria	Yes
**Score (of 8)**	62.5

Mir-93	Ma et al., 2019 (63)
Prospective	Yes
Biomarker evaluator blinded to outcome	yes
Defined time period	No
Pre-defined clinical endpoints	Yes
Sample size calculation	No
Candidate variables	No
Measurement method described	Yes
Inclusion/exclusion criteria	Yes
**Score (of 8)**	62.5

Supplemental References

1. Xiang W, Tian C, Lin J, Wu X, Pang G, Zhou L, et al. Plasma let-7i and miR-15a expression are associated with the effect of recombinant tissue plasminogen activator treatment in acute ischemic stroke patients. Thromb Res. octombrie 2017;158:121–5.

2. Wang ZQ, Li K, Huang J, Huo TT, Lv PY. MicroRNA Let-7i Is a Promising Serum Biomarker for Post-stroke Cognitive Impairment and Alleviated OGD-Induced Cell Damage in vitro by Regulating Bcl-2. Front Neurosci. 24 martie 2020;14:215.

3. Jickling GC, Ander BP, Shroff N, Orantia M, Stamova B, Dykstra-Aiello C, et al. Leukocyte response is regulated by microRNA let7i in patients with acute ischemic stroke. Neurology. 22 noiembrie 2016;87(21):2198–205.

4. Wang J, Huang Q, Ding J, Wang X. Elevated serum levels of brain-derived neurotrophic factor and miR-124 in acute ischemic stroke patients and the molecular mechanism. 3 Biotech. noiembrie 2019;9(11):386.

5. Yuan M, Guo YS, Zhang XX, Gao ZK, Shen XY, Han Y, et al. Diagnostic performance of miR-21, miR-124, miR-132, and miR-200b serums in post-stroke cognitive impairment patients. Folia Neuropathol. 2022;60(2):228–36.

6. Xie Z, Liu B, Zhou M, Chen Y. Expression and clinical significance of plasma miR-124 in acute ischemic stroke. The Journal of Practical Medicine. 2019;343–5.

7. Zhou X, Qi L. miR-124 Is Downregulated in Serum of Acute Cerebral Infarct Patients and Shows Diagnostic and Prognostic Value. Clin Appl Thromb Hemost. 26 octombrie 2021;27:10760296211035446.

8. Ji Q, Ji Y, Peng J, Zhou X, Chen X, Zhao H, et al. Increased Brain-Specific MiR-9 and MiR-124 in the Serum Exosomes of Acute Ischemic Stroke Patients. PLoS One. 2016;11(9):e0163645.

9. He XW, Shi YH, Liu YS, Li GF, Zhao R, Hu Y, et al. Increased plasma levels of miR-124-3p, miR-125b-5p and miR-192-5p are associated with outcomes in acute ischaemic stroke patients receiving thrombolysis. Atherosclerosis. octombrie 2019;289:36–43.

10. Qi Z, Zhao Y, Su Y, Cao B, Yang JJ, Xing Q. Serum Extracellular Vesicle–Derived miR-124-3p as a Diagnostic and Predictive Marker for Early-Stage Acute Ischemic Stroke. Frontiers in Molecular Biosciences [Internet]. 2021 [citat 2 septembrie 2022];8. Disponibil la: https://www.frontiersin.org/articles/10.3389/fmolb.2021.685088

11. Jin F, Xing J. Circulating pro-angiogenic and anti-angiogenic microRNA expressions in patients with acute ischemic stroke and their association with disease severity. Neurol Sci. noiembrie 2017;38(11):2015–23.

12. Jin F, Xing J. Circulating miR-126 and miR-130a levels correlate with lower disease risk, disease severity, and reduced inflammatory cytokine levels in acute ischemic stroke patients. Neurol Sci. octombrie 2018;39(10):1757–65.

13. Chen X, Zhang X, Su C, Huang S. Long noncoding RNA HULC in acute ischemic stroke: Association with disease risk, severity, and recurrence-free survival and relation with IL-6, ICAM1, miR-9, and miR-195. J Clin Lab Anal. 20 august 2020;34(11):e23500.

14. Yang G, Liu Z, Wang L, Chen X, Wang X, Dong Q, et al. MicroRNA-195 protection against focal cerebral ischemia by targeting CX3CR1. J Neurosurg. 1 noiembrie 2018;1–10.

15. Zhai Y, Zhu Z, Li H, Zhao C, Huang Y, Wang P. miR-195 and miR-497 in acute stroke and their correlations with post-stroke cognitive impairment. Int J Clin Exp Pathol. 1 decembrie 2020;13(12):3092–9.

16. Liu C, Huang H, Li Y, Zhao H. The relationship of long non-coding RNA maternally expressed gene 3 with microRNA-21 and their correlation with acute ischemic stroke risk, disease severity and recurrence risk. Clin Neurol Neurosurg. noiembrie 2021;210:106940.

17. Zhou J, Zhang J. Identification of miRNA-21 and miRNA-24 in plasma as potential early stage markers of acute cerebral infarction. Mol Med Rep. august 2014;10(2):971–6.

18. Wang Y, Huang J, Ma Y, Tang G, Liu Y, Chen X, et al. MicroRNA-29b is a therapeutic target in cerebral ischemia associated with aquaporin 4. J Cereb Blood Flow Metab. decembrie 2015;35(12):1977–84.

19. Guo C, Zhong C, Li Q, Gao Y, Li W, Ou Y. [Expressions and neural function prognostic evaluation of serum microRNA-24 and microRNA-29b in elderly patients with acute ischemic stroke]. Zhonghua Wei Zhong Bing Ji Jiu Yi Xue. ianuarie 2020;32(1):78–82.

20. Ma X, Yun HJ, Elkin K, Guo Y, Ding Y, Li G. MicroRNA-29b Suppresses Inflammation and Protects Blood-Brain Barrier Integrity in Ischemic Stroke. Mediators of Inflammation. 23 august 2022;2022:e1755416.

21. Li G, Ma X, Zhao H, Fan J, Liu T, Luo Y, et al. Long non-coding RNA H19 promotes leukocyte inflammation in ischemic stroke by targeting the miR-29b/C1QTNF6 axis. CNS Neuroscience & Therapeutics. 2022;28(6):953–63.

22. Abdelaleem OO, Shaker OG, Mohamed MM, Ahmed TI, Elkhateeb AF, Abdelghaffar NK, et al. Differential Expression of Serum TUG1, LINC00657, miR-9, and miR-106a in Diabetic Patients With and Without Ischemic Stroke. Frontiers in Molecular Biosciences [Internet]. 2022 [citat 31 august 2022];8. Disponibil la: https://www.frontiersin.org/articles/10.3389/fmolb.2021.758742

23. Xue Y, Li M, Liu D, Zhu Q, Chen H. Expression of miR-9 in the serum of patients with acute ischemic stroke and its effect on neuronal damage. Int J Clin Exp Pathol. 2018;11(12):5885–92.

24. Kautzky V. Identifizierung von Einflussfaktoren auf die Konzentrationen der zirkulierenden microRNAs miR-125a-5p, miR-125b-5p und miR-143-3p nach ischämischem Schlaganfall [Internet] [Text.PhDThesis]. Ludwig-Maximilians-Universität München; 2022 [citat 9 octombrie 2022]. Disponibil la: https://edoc.ub.uni-muenchen.de/29704/

25. He XW, Shi YH, Zhao R, Liu YS, Li GF, Hu Y, et al. Plasma Levels of miR-125b-5p and miR-206 in Acute Ischemic Stroke Patients After Recanalization Treatment: A Prospective Observational Study. J Stroke Cerebrovasc Dis. iunie 2019;28(6):1654–61.

26. Yuan M, Yuan H, Zhou C, Liu F, Lin C, Tang Y. The significance of low plasma miR-335 level in patients with acute cerebral infarction may be associated with the loss of control of CALM1 expression. Int J Clin Exp Med. 2016;9(10):19595–601.

27. Zhao B, Zhu Z, Hao J, Wan Z, Guo X. Decreased plasma miR-335 expression in patients with acute ischemic stroke and its association with calmodulin expression. J Int Med Res. decembrie 2016;44(6):1331–8.

28. Guo C, Yao Y, Li Q, Gao Y, Cao H. Expression and Clinical Value of miR-185 and miR-424 in Patients with Acute Ischemic Stroke. Int J Gen Med. 4 ianuarie 2022;15:71–8.

29. Kijpaisalratana N, Nimsamer P, Khamwut A, Payungporn S, Pisitkun T, Chutinet A, et al. Serum miRNA125a-5p, miR-125b-5p, and miR-433-5p as biomarkers to differentiate between posterior circulation stroke and peripheral vertigo. BMC Neurology. 10 octombrie 2020;20(1):372.

30. Ma X, Liao X, Liu J, Wang Y, Wang X, Chen Y, et al. Circulating endothelial microvesicles and their carried miR-125a-5p: potential biomarkers for ischaemic stroke. Stroke Vasc Neurol. 15 septembrie 2022;svn.

31. Peng G, Yuan Y, Wu S, He F, Hu Y, Luo B. MicroRNA let-7e Is a Potential Circulating Biomarker of Acute Stage Ischemic Stroke. Transl Stroke Res. decembrie 2015;6(6):437–45.

32. Wu J, Fan CL, Ma LJ, Liu T, Wang C, Song JX, et al. Distinctive expression signatures of serum microRNAs in ischaemic stroke and transient ischaemic attack patients. Thromb Haemost. 3 mai 2017;117(5):992–1001.

33. Zeng L, Liu J, Wang Y, Wang L, Weng S, Chen S, et al. Cocktail blood biomarkers: prediction of clinical outcomes in patients with acute ischemic stroke. Eur Neurol. 2013;69(2):68–75.

34. Otero-Ortega L, Alonso-López E, Pérez-Mato M, Laso-García F, Gómez-de Frutos MC, Diekhorst L, et al. Circulating Extracellular Vesicle Proteins and MicroRNA Profiles in Subcortical and Cortical-Subcortical Ischaemic Stroke. Biomedicines. 7 iulie 2021;9(7):786.

35. Yang ZB, Li TB, Zhang Z, Ren KD, Zheng ZF, Peng J, et al. The Diagnostic Value of Circulating Brain-specific MicroRNAs for Ischemic Stroke. Intern Med. 2016;55(10):1279–86.

36. Zhu X, Ding J, Wang B, Wang J, Xu M. Circular RNA DLGAP4 is down-regulated and negatively correlates with severity, inflammatory cytokine expression and pro-inflammatory gene miR-143 expression in acute ischemic stroke patients. Int J Clin Exp Pathol. 1 martie 2019;12(3):941–8.

37. Niu M, Li H, Li X, Yan X, Ma A, Pan X, et al. Circulating Exosomal miRNAs as Novel Biomarkers Perform Superior Diagnostic Efficiency Compared With Plasma miRNAs for Large-Artery Atherosclerosis Stroke. Front Pharmacol. 26 noiembrie 2021;12:791644.

38. Huang S, Zhao J, Huang D, Zhuo L, Liao S, Jiang Z. Serum miR-132 is a risk marker of post-stroke cognitive impairment. Neurosci Lett. 26 februarie 2016;615:102–6.

39. Lin X, Wang W, Tao T, Zhang D, Mao L, He X. Synthetic role of miR-411-5p and CT perfusion information in predicting clinical outcomes after thrombolysis in acute cerebral infarction. Acta Neurol Belg. 6 august 2022;

40. Yuan M, Tang Y, Zhou C, Liu F, Chen L, Yuan H. Elevated plasma CaM expression in patients with acute cerebral infarction predicts poor outcomes and is inversely associated with miR-26b expression. Int J Neurosci. 2016;126(5):408–14.

41. Zhong C, Yin C, Niu G, Ning L, Pan J. MicroRNA miR-497 is closely associated with poor prognosis in patients with cerebral ischemic stroke. Bioengineered. 12(1):2851–62.

42. Song XD, Li SX, Zhu M. Plasma miR-409-3p promotes acute cerebral infarction via suppressing CTRP3. Kaohsiung J Med Sci. aprilie 2021;37(4):324–33.

43. Zhang H, Chen G, Qiu W, Pan Q, Chen Y, Chen Y, et al. Plasma endothelial microvesicles and their carrying miRNA-155 serve as biomarkers for ischemic stroke. J Neurosci Res. noiembrie 2020;98(11):2290–301.

44. Yang S, Zhan X, He M, Wang J, Qiu X. miR-135b levels in the peripheral blood serve as a marker associated with acute ischemic stroke. Exp Ther Med. iunie 2020;19(6):3551–8.

45. Liu P, Han Z, Ma Q, Liu T, Wang R, Tao Z, et al. Upregulation of MicroRNA-128 in the Peripheral Blood of Acute Ischemic Stroke Patients is Correlated with Stroke Severity Partially through Inhibition of Neuronal Cell Cycle Reentry. Cell Transplant. iulie 2019;28(7):839–50.

46. Zhao H, Li G, Wang R, Tao Z, Ma Q, Zhang S, et al. Silencing of microRNA-494 inhibits the neurotoxic Th1 shift via regulating HDAC2-STAT4 cascade in ischaemic stroke. Br J Pharmacol. ianuarie 2020;177(1):128–44.

47. Sheikhbahaei S, Manizheh D, Mohammad S, Hasan TM, Saman N, Laleh R, et al. Can MiR-503 be used as a marker in diabetic patients with ischemic stroke? BMC Endocr Disord. 29 aprilie 2019;19(1):42.

48. Fu C, Chen S, Cai N, Liu Z, Wang P, Zhao J. Potential Neuroprotective Effect of miR-451 Against Cerebral Ischemia/Reperfusion Injury in Stroke Patients and a Mouse Model. World Neurosurg. octombrie 2019;130:e54–61.

49. Ye Z, Hu J, Xu H, Sun B, Jin Y, Zhang Y, et al. Serum Exosomal microRNA-27-3p Aggravates Cerebral Injury and Inflammation in Patients with Acute Cerebral Infarction by Targeting PPARγ. Inflammation. iunie 2021;44(3):1035–48.

50. Zhou J, Chen L, Chen B, Huang S, Zeng C, Wu H, et al. Increased serum exosomal miR-134 expression in the acute ischemic stroke patients. BMC Neurol. 4 decembrie 2018;18:198.

51. Chen Z, Wang K, Huang J, Zheng G, Lv Y, Luo N, et al. Upregulated Serum MiR-146b Serves as a Biomarker for Acute Ischemic Stroke. Cell Physiol Biochem. 2018;45(1):397–405.

52. Chen Y, Song Y, Huang J, Qu M, Zhang Y, Geng J, et al. Increased Circulating Exosomal miRNA-223 Is Associated with Acute Ischemic Stroke. Front Neurol. 2017;8:57.

53. Wang Y, Zhang Y, Huang J, Chen X, Gu X, Wang Y, et al. Increase of circulating miR-223 and insulin-like growth factor-1 is associated with the pathogenesis of acute ischemic stroke in patients. BMC Neurol. 8 aprilie 2014;14:77.

54. Liang T ying, Lou J yu. Increased Expression of mir-34a-5p and Clinical Association in Acute Ischemic Stroke Patients and in a Rat Model. Med Sci Monit. 22 august 2016;22:2950–5.

55. Wang Q, Wang F, Fu F, Liu J, Sun W, Chen Y. Diagnostic and prognostic value of serum miR-9-5p and miR-128-3p levels in early-stage acute ischemic stroke. Clinics (Sao Paulo). 2021;76:e2958.

56. Jia L, Hao F, Wang W, Qu Y. Circulating miR-145 is associated with plasma high-sensitivity C-reactive protein in acute ischemic stroke patients. Cell Biochemistry and Function. 2015;33(5):314–9.

57. Liang HB, He JR, Tu XQ, Ding KQ, Yang GY, Zhang Y, et al. MicroRNA-140-5p: A novel circulating biomarker for early warning of late-onset post-stroke depression. J Psychiatr Res. august 2019;115:129–41.

58. Hu J, Zhou W, Zhou Z, Yang Q, Xu J, Dong W. miR-22 and cerebral microbleeds in brainstem and deep area are associated with depression one month after ischemic stroke. Braz J Med Biol Res. 27 aprilie 2020;53(5):e9162.

59. Cui Y, Ma G, Kong F, Song L. Diagnostic Values of miR-221-3p in Serum and Cerebrospinal Fluid for Post-Stroke Depression and Analysis of Risk Factors. Iranian Journal of Public Health. iunie 2021;50(6):1241.

60. Zeng L, Liu J, Wang Y, Wang L, Weng S, Tang Y, et al. MicroRNA-210 as a novel blood biomarker in acute cerebral ischemia. Front Biosci (Elite Ed). 1 iunie 2011;3:1265–72.

61. Kotb HG, Ibrahim AH, Mohamed EF, Ali OM, Hassanein N, Badawy D, et al. The expression of microRNA 146a in patients with ischemic stroke: an observational study. Int J Gen Med. 9 august 2019;12:273–8.

62. Wu X, Zhang X, Li D, Zhu Z. Plasma level of miR-99b may serve as potential diagnostic and short-term prognostic markers in patients with acute cerebral infarction. J Clin Lab Anal. 22 ianuarie 2020;34(3):e23093.

63. Ma Q, Li G, Tao Z, Wang J, Wang R, Liu P, et al. Blood microRNA-93 as an indicator for diagnosis and prediction of functional recovery of acute stroke patients. J Clin Neurosci. aprilie 2019;62:121–7.
